# Discovery of Biofilm Inhibitors from the Microbiota of Marine Egg Masses

**DOI:** 10.1021/acs.jnatprod.4c00376

**Published:** 2024-05-30

**Authors:** Lois Kyei, Karla Piedl, Carla Menegatti, Eleanor M. Miller, Emily Mevers

**Affiliations:** † Department of Chemistry, Virginia Tech, Blacksburg, Virginia 24061, United States

## Abstract

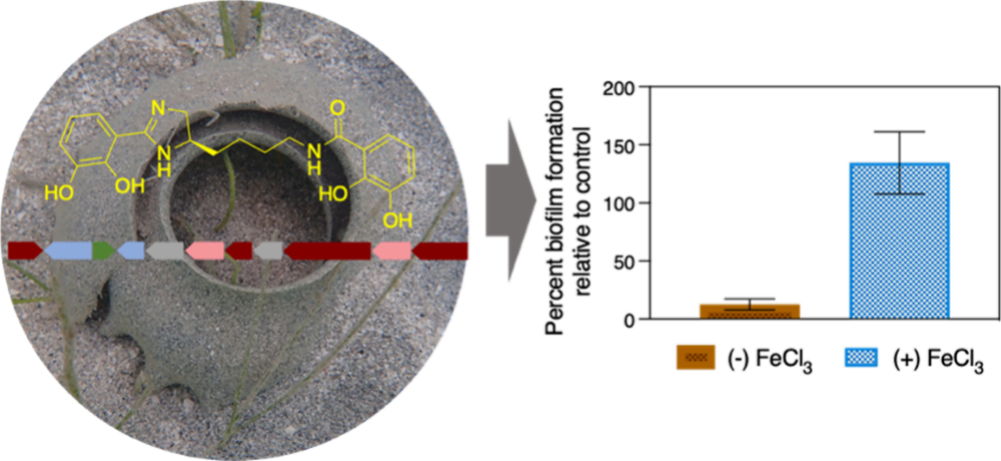

Biofilms commonly develop in immunocompromised patients, which leads to persistent infections that are difficult to treat. In the biofilm state, bacteria are protected against both antibiotics and the host’s immune system; currently, there are no therapeutics that target biofilms. In this study, we screened a chemical fraction library representing the natural product capacity of the microbiota of marine egg masses, namely, the moon snail egg collars. This led to the identification of active fractions targeting both *Pseudomonas aeruginosa* and *Staphylococcus aureus* biofilms. Subsequent analysis revealed that a subset of these fractions were capable of eradicating preformed biofilms, all against *S. aureus*. Bioassay-guided isolation led us to identify pseudochelin A, a known siderophore, as a *S. aureus* biofilm inhibitor with an IC_50_ of 88.5 μM. Mass spectrometry-based metabolomic analyses revealed widespread production of pseudochelin A among fractions possessing *S. aureus* antibiofilm properties. In addition, a key biosynthetic gene involved in producing pseudochelin A was detected on 30% of the moon snail egg collars and pseudochelin A is capable of inhibiting the formation of biofilms (IC_50_ 50.6 μM) produced by ecologically relevant bacterial strains. We propose that pseudochelin A may have a role in shaping the microbiome or protecting the egg collars from microbiofouling.

Antibiotic resistance has rendered many conventional antibiotics obsolete and contributed to the deaths of 1.2 million people in 2019.^1^ The ESKAPE pathogens—*Enterococcus faecium*, *Staphylococcus aureus*, *Klebsiella pneumoniae*, *Acinetobacter baumannii*, *Pseudomonas aeruginosa*, and *Enterobacter* sp.—pose the largest challenges as they contain multiple resistance mechanisms, surviving up to 10–1000-fold the normal doses of antibiotics.^2,3^ In addition, several ESKAPE pathogens actively evade antibiotics by forming protective barriers known as biofilms.^4^ Biofilms, surface-adhered multicellular communities of bacteria, are enclosed in a matrix of extracellular polymeric substances. Bacteria within biofilms can also tolerate up to 1000-fold the normal dose of antibiotics.^5^ Biofilm infections contribute to approximately 80% of all chronic bacterial infections with ESKAPE-pathogen biofilm infection posing a significant challenge with current antibiotic therapies.^6^

Although an obvious need exists for small molecule therapeutics targeting biofilm infections, the FDA has approved none. Efforts to directly target biofilms with small molecules have focused on developing therapeutics that disrupt biofilm formation or eradicate already formed biofilms. These therapeutics would be used in conjunction with traditional antibiotics to eliminate the infection completely.^7^ In recent years, several natural products have been shown to possess the desired activity in either *in vitro* or *in vivo* assays.^8,9^ A couple of examples include auromomycin, which is produced by a marine sediment bacterium and strongly inhibits biofilm formation (IC_50_ 60.1 μM) by *Vibrio cholerae*.^10^ A subsequent structure–activity relationship study led to a related oxazine that is 10-fold more potent (IC_50_ 6 μM) while also possessing biofilm disruption properties.^11^ In addition, two marine sponges, *Agelas conifera* and *A. oroides*, produce bromoageliferin and oroidin, bromopyrrole alkaloids that inhibit biofilm formation in methicillin-resistant *S. aureus* and *A. baumannii*.^12−14^ Finally, streptorubin B, produced by multiple *Streptomyces* strains, inhibits biofilm formation in *S. aureus* by at least 70% at about 1 μg/mL.^15^ The mechanism of action of many biofilm inhibitors/disruptors is unknown. However, several hypotheses abound, including that they disrupt quorum sensing,^16,17^ chelate iron,^18^ or target the cyclic di-GMP signaling pathway.^7,8,19^

Innate substrates in the marine environment become microbiofouled, accumulating microorganisms on wet surfaces, along with many living substrates, such as mangrove roots and seagrass.^20,21^ Microbiofouling is mechanistically similar to forming biofilms in humans.^9^ Interestingly, some marine organisms appear to resist biofouling; these organisms can be leveraged as a source of potential antibiofilm metabolites.^22,23^ One particular source is marine egg masses or clutches. Many marine organisms lay egg masses that appear to be unprotected (i.e., they have no hard external shell or parental protection) yet, they do not seem to experience biofouling.^24^ This includes egg masses of the Hawaiian Bobtail Squid,^25^ moon snails,^26,27^ and nudibranchs.^28^ Researchers have proposed that the microbiota of egg masses has a role in preventing microbial fouling, as treatment with antibiotics prior to microbial challenge experiments renders the egg masses more sensitive to infection.^29,30^

To identify bacterial natural products that inhibit formation of or disrupt preformed biofilms from *P. aeruginosa* and *S. aureus,* we screened a library of 810 chemical fractions representing 140 bacterial strains isolated primarily from moon snail egg collars for biofilm inhibition properties. Although several fractions inhibited biofilm formation in *S. aureus* and *P. aeruginosa*, only two inhibited biofilm formation in both organisms. Mass spectrometry (MS)-based metabolomics and bioassay-guided isolation led us to identify pseudochelin A (**1**), a known siderophore,^31^ as the active metabolite in 24 fractions possessing biofilm inhibition activity against *S. aureus*. In addition, **1** was observed to inhibit the formation of biofilms produced by ecologically relevant bacteria, and a key biosynthetic gene responsible for producing **1** was amplified from DNA samples of single moon snail egg collars. Consequently, we propose that **1** may have an ecological role in shaping the microbiome and/or protecting the egg collars from biofouling.

## Results and Discussion

As part of our ongoing effort to identify natural products with antimicrobial properties from the microbiota of marine egg masses, we have assembled over 800 bacterial strains from various marine egg masses. These strains, isolated primarily from moon snail egg collars collected in Florida and Puerto Rico, also contain bacterial strains from egg masses of various gastropods (Table S1). A subset of these bacterial strains was grown under three media conditions (1 L per condition) with hydrophobic resins that captured secreted bacterial secondary metabolites. Extracting the resins and subsequent fractionation using flash chromatography (C8 resin) yielded eight chemically distinct fractions per bacterial strain. Individual fractions (810) were resuspended at 10 mg/mL in DMSO and arrayed into 96-well plates.

**Figure 1:**
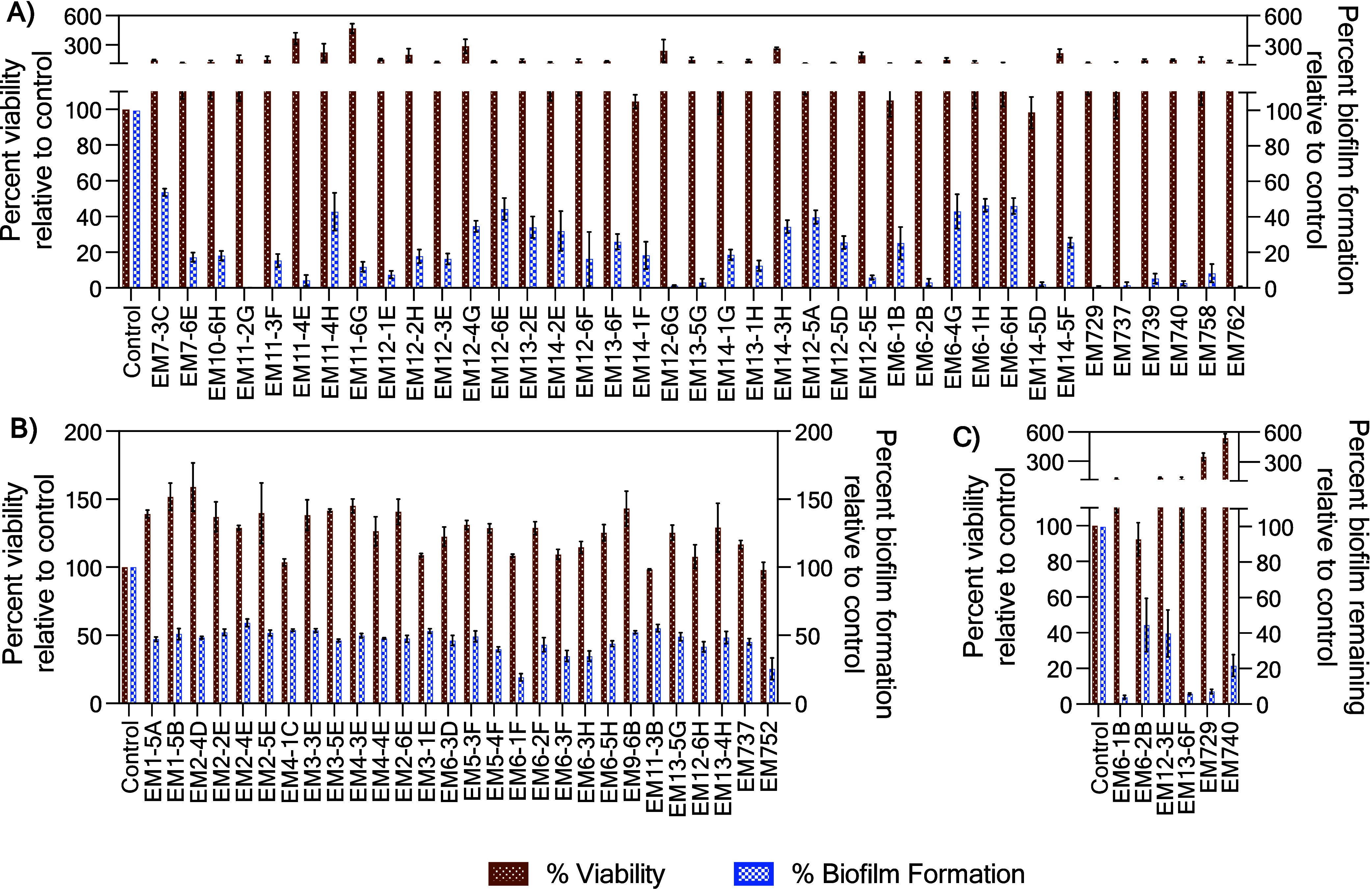
Fraction library was screened for biofilm inhibition and disruption properties. **(A)** Thirty-nine fractions inhibited >50% of biofilm formation against *S. aureus* without negatively impacting growth viability, **(B)** twenty-eight fractions inhibited >50% of biofilm formation against *P. aeruginosa* without negatively impacting growth viability, and **(C)** six fractions disrupted preformed *S. aureus* biofilms.

During the collection of the moon snail egg collars, field observations suggested that the egg collars were less prone to biofouling than other substrates (shells and seagrass). This led to our hypothesis that the microbiota of these egg collars chemically defends the eggs from microbiofouling. To test this, we screened the microbial fraction library for biofilm inhibition and disruption against two pathogens, *S. aureus* and *P. aeruginosa.* First, we screened for biofilm inhibition: each fraction was incubated overnight with the pathogen, plates were rinsed to remove planktonic cells, and residual biofilms were stained with crystal violet for quantification (see experimental methods). In total, thirty-nine and twenty-eight fractions inhibited >50% of *S. aureus* (4.8% hit rate) and *P. aeruginosa* (3.5% hit rate) biofilm formation, respectively (Figure 1A and 1B). The phylogenetic diversity of the active fractions was highly similar among the active fractions for each pathogen, with a 1:1 ratio of the active fractions being produced by Gram-positive versus Gram-negative strains. In addition, ∼36% of the active fractions were produced by Bacilli, ∼31% by Gammaproteobacteria, ∼13% by Actinomycetia, ∼10% by Alphaproteobacteria, and ∼10% by Flavobacteriia (Table S1). However, despite the high phylogenetic similarity of the producing organisms, there was minimal overlap in the actual active fractions, with only two fractions (EM13–5 and EM737 produced by *Aquimarina* sp. and *Staphylococcus* sp., respectively) exhibiting biofilm inhibition activity against both *S. aureus* and *P. aeruginosa*.

Second, we screened all fractions that exhibited biofilm inhibition for biofilm disruption properties. Biofilms for each pathogen were preformed and then treated with each fraction. The biofilms were incubated with the fraction overnight, washed, and the remaining biofilm quantified. Only six fractions disrupted >50% of *S. aureus* biofilms (15.4% of the biofilm inhibitors), while no fraction significantly disrupted *P. aeruginosa* biofilms (Figure 1C). The fractions that disrupted *S. aureus* biofilms were produced by Bacilli (three), Actinomycetia (two) and Flavobacteriia (one).

To identify the small molecules responsible for the observed biofilm inhibition and disruption, we chose a medium polarity fraction (fraction E that eluted at 60% MeOH/Water) produced by *Pseudoalteromonas piscicida* EM138 (EM11–4). This particular fraction inhibited 96% of biofilm formation in *S. aureus* without impacting viability. High performance liquid chromatography (HPLC) under reversed phase conditions led to the isolation of pseudochelin A (**1**). Compound **1** is a siderophore previously purified from *P. piscicida* S2040 isolated from sediment off the northwestern coast of Australia.^31^ We confirmed the structure of **1** by comparing HR-ESI-MS, 1D and 2D NMR, and ECD spectra to published data (Figure S1–S6).
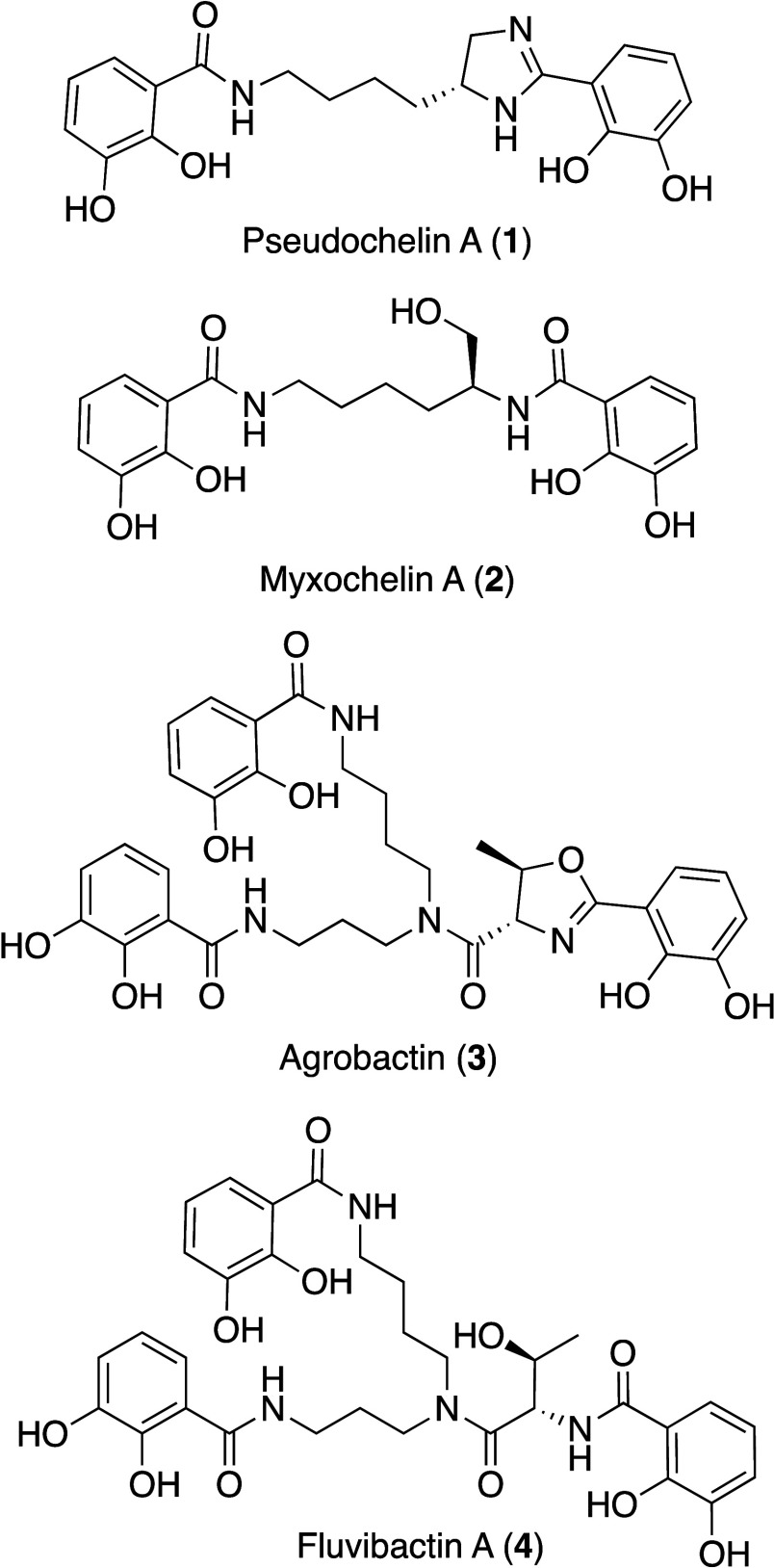


**Figure 2:**
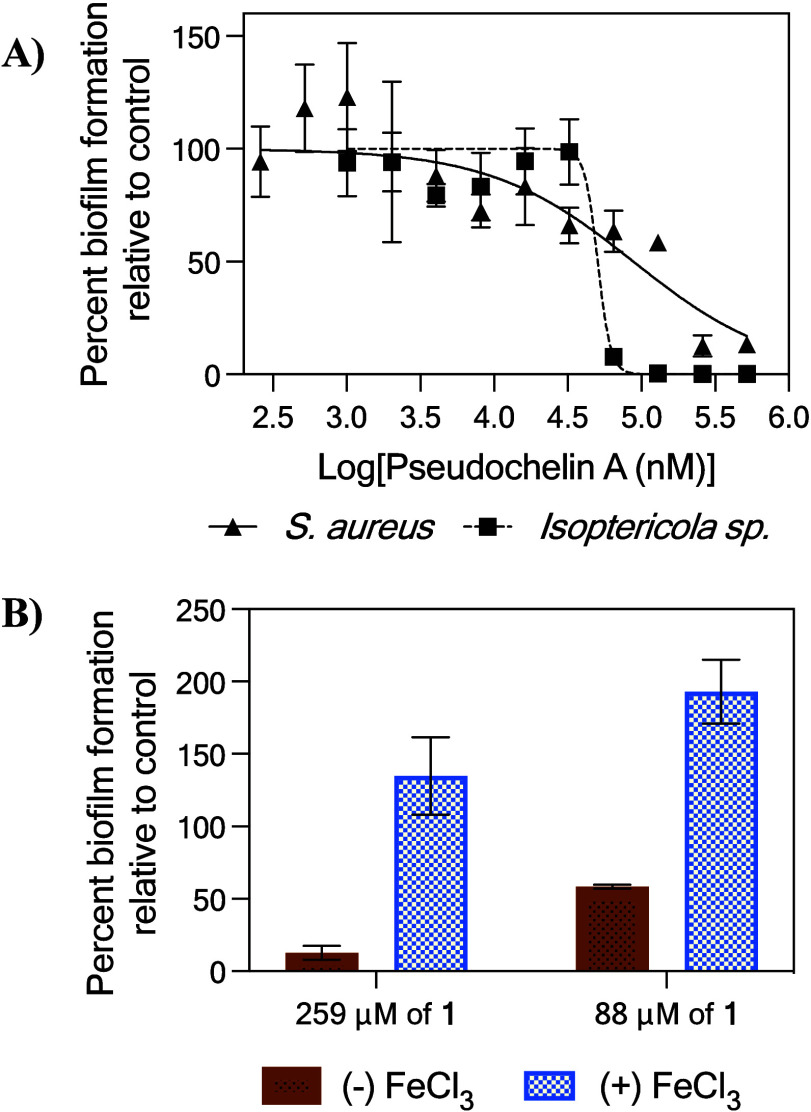
Biological activity for pseudochelin A. **(A)** Dose-dependent biofilm inhibition of **1** against *S. aureus* (triangles and solid line) and *Isoptericola* sp. (squares and dotted line), and **(B)** Impact of FeCl_3_ (12.5 μM) on the biofilm inhibition activity by **1** against *S. aureus*. Compound **1** was evaluated for biofilm inhibition with (blue bars) and without (brown bars) FeCl_3_.

We evaluated purified **1** for both biofilm inhibition and disruption properties against *S. aureus*. Compound **1** inhibited biofilm formation in a dose-dependent fashion with an IC_50_ of 88.5 μM with no impact on overall growth of the pathogen (Figure 2A and Figure S7) and exhibited only minimal biofilm disruption activity (30% disruption at 518 μM - Figure S8). Compound **1** was not evaluated against *P. aeruginosa* because the crude fraction containing **1** was inactive. Although **1** impacts biofilm-formation in *S. aureus*, this is not ecologically relevant as *S. aureus* is not commonly found in the marine environment. Therefore, we investigated the ability of **1** to suppress biofilm growth of bacterial strains found associated with moon snail egg collars. Compound **1** was screened against five biofilm forming bacterial strains that were isolated from the same source as *P. piscicida* EM138, representing three genera: *Bacillus*, *Isoptericola*, and *Labrenzia* (Figure S9). Compound **1** inhibited biofilm growth against the two *Isoptericola* strains (Class: Actinomycetia), with an IC_50_ of 50.6 μM against one of the *Isoptericola* strains (Figure 2A). Thereby, suggesting production of **1** could afford competitive advantage to the producing organism through suppression of biofilm formation by other bacterial strains.

Siderophores are known to inhibit biofilm formation through sequestration of iron,^32^ therefore we presumed that the affinity of **1** for Fe^3+^ was responsible for its biofilm inhibition property. Iron, an essential nutrient, is critical to many life processes, including bacterial biofilm formation.^18,33^ Under physiological conditions, iron is mostly in its insoluble form (Fe^3+^) and not readily bioavailable.^34^ Hence, siderophores have evolved to have high affinity for Fe^3+^ to capture this essential element.^35^ We hypothesized that **1** inhibits biofilm formation in *S. aureus* by reducing the overall concentration of soluble Fe^3+^. Therefore, increasing the media iron concentration should curtail the biofilm inhibition activity of **1**. Previous work by Greenberg and colleagues supports this hypothesis as increased concentration of available iron serves as a signal for biofilm development in *P. aeruginosa*.^36^ We tested this hypothesis by repeating the biofilm formation assay with *S. aureus* in media supplemented with and without FeCl_3_ (various concentrations). Iron concentrations above 6.25 μM abolished the antibiofilm activity of **1** (Figure 2B and Figure S10), indicating that **1** is impacting *S. aureus* biofilm by reducing overall Fe^3+^ bioavailability. Interestingly, under our assay conditions, *S. aureus* biofilms are inhibited by FeCl_3_ concentrations >12.5 μM (Figure S11).

**Figure 3:**
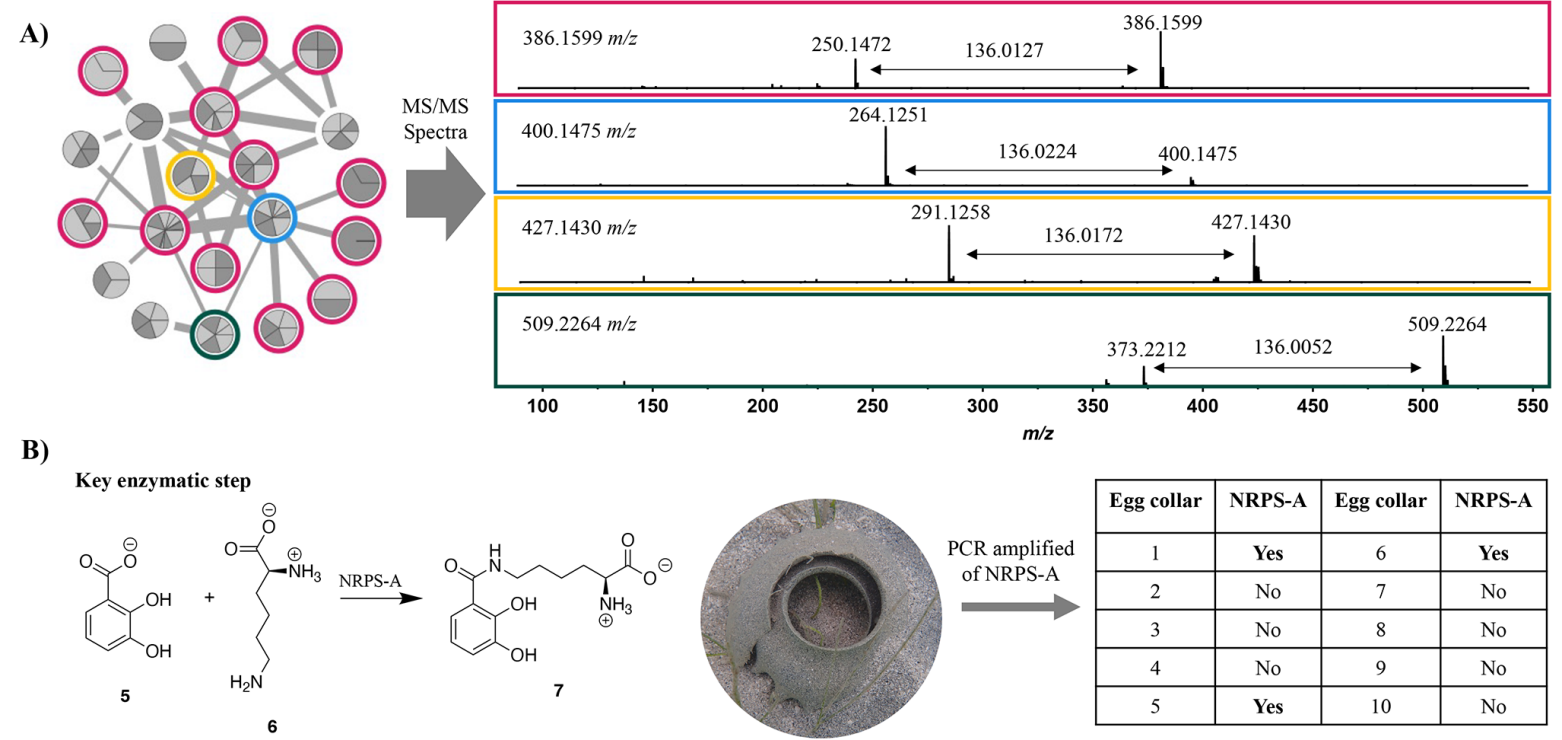
Widespread production of pseudochelin A. **(A)** GNPS cluster of catechol-containing siderophores where node pie shading represents number of producing bacteria (Table S2) and edge thickness represents cosine score. Examples of fragmentation patterns from four distinct highlighted nodes with the diagnostic 136 Da neutral loss, and **(B)** Amplification of the NRPS adenylation domain, which performs a key enzymatic step in the biosynthesis of pseudochelin A was used to detect the biosynthetic gene cluster (BGC) of **1** within the moon collar microbiota of environmental samples.

Pseudochelin A belongs to a larger family of catechol-containing natural product siderophores, including the myxochelins (**2**),^37^ agrobactins (**3**),^38^ and fluvibactins (**4**).^39^ Each is biosynthesized by a hybrid nonribosomal peptide synthetase (NRPS) and shikimate pathway, and all contain two or more 2,3-dihydroxybenzoates (2,3-DHB).^37^ When this family of metabolites is analyzed via tandem MS, they yield a diagnostic neutral loss of 136 Da (**1**, 386 → 250 *m*/*z*; **2**, 405 → 269 *m*/*z*; **3**, 637 → 501 *m*/*z*; **4**, 655 → 519 *m*/*z*), which is representative of the cleavage of the amide bond, liberating 2,3-DHB (**5**). Using this key fragmentation pattern, we analyzed the LCMS data for all fractions that inhibited *S. aureus* biofilm formation for the presence of these metabolites. Overall, 24 fractions contained **1** (62% of the active fractions), representing 16 bacterial strains. A wider metabolomic analysis using Global Natural Product Social (GNPS) molecular networking was used to analyze tandem MS data representing all fractions in the microbial fraction library [fractions were pooled based on polarity (A–D and E–H)].^40^ Node pie shading represents the number of bacterial strains that possess the same precursor mass (Figure 3A and Table S2). Overall, 16 bacterial strains produced **1** or a related analog—ten of these strains inhibited biofilm formation in *S. aureus*, five exhibited antimicrobial activity, and one strain did not possess either activity. Eleven precursor masses contained a neutral loss of 136 Da (Figure S12–S22), including **1**, and clustered together when analyzed with a cosine score of 0.65 (Figure 3A). Dereplication of the other precursor masses clustering with **1** by using NPAtlas and AntiBase did not lead to any matches.^41^

Given the large number of bacterial strains producing catechol-containing siderophores within our relatively small microbial fraction library, it is likely that these molecules are also produced on or within the egg masses. A bulk collection of moon snail egg collars collected in Feb. 2023 was extracted and fractionated using a similar purification scheme as the microbial crude extracts. Although these fractions exhibited some biofilm inhibition against *S. aureus* (Figure S23), MS-based metabolomics failed to reveal catechol-containing siderophores.

Instead of detecting the actual metabolite, we asked whether individual egg collars in SW Florida contained the biosynthetic gene cluster (BGC) of **1**. During the Feb. 2023 collection trip, we systematically collected moon snail egg collars at the same sites as our previous collections. Ten individual moon snail egg collars were collected at each site, preserved in ethanol, and the environmental DNA extracted. Analysis of the sequenced *P. piscicida* EM138 genome in AntiSMASH^42^ identified a putative BGC for **1**, which had 100% homology to previously reported pathways.^37^ We designed PCR primers for a key biosynthetic gene, specifically for the NRPS adenylation domain (NRPS-A), which forms the amide bond between **5** and lysine (**6**) (Figure 3B). We used this set of primers to amplify the gene from the extracted egg collar DNA using PCR. Interestingly, the NRPS-A was observed in three of the ten samples (Figure S24). The presence of this gene was confirmed by Illumina sequencing of the PCR product (Table S4), thus strongly suggesting that 1) the microbiota of the moon snail egg collars have the potential to make **1** under the right conditions and 2) this molecule may have a role in shaping the composition of the host’s microbiota.

In summary, this study begins to establish the microbiota of marine egg masses, specifically those from moon snail egg collars, as a source of metabolites with antibiofilm properties. We have identified the active metabolite, pseudochelin A, as the responsible agent for biofilm-formation inhibition in *S. aureus* and its presence in 24 of the thirty-eight active fractions from extracts of bacteria in our strain library. Pseudochelin A, a known siderophore, has biofilm-inhibition properties that appear to be associated with its iron-sequestration properties. In addition, a key biosynthetic gene was amplified from DNA extractions of 30% of the collected egg collars. Thus, we suggest that pseudochelin A may have an ecological role in protecting the egg collars from biofouling. Work is ongoing to identify the metabolites responsible for eradicating preformed biofilms and those that inhibit *P. aeruginosa* biofilms.

## Experimental Section

### General Experimental Procedures

ECD and UV/vis spectra were recorded on a JASCO J-815 spectrophotometer. NMR spectra were recorded in deuterated acetone with the residual solvent peak as an internal standard (δ_C_ 28.97, δ_H_ 2.05) on a Bruker Avance III 600 MHz instrument equipped with a triple resonance inverse (CP-TCI) Prodigy N2 cooled CryoProbe (600 and 150 MHz for ^1^H and ^13^C NMR, respectively). LR-LCMS data was obtained on an Agilent 1200 series HPLC system equipped with a photodiode array detector and a Thermo LTQ mass spectrometer. HR-ESI-MS was carried out on either a Shimadzu q-ToF mass spectrometer equipped with an HPLC system or an Agilent 6530 q-ToF equipped with a 1290 Infinity II UPLC system. HPLC purifications were carried out on Agilent 1200 series or 1260 Infinity II HPLC systems (Agilent Technologies) equipped with a photodiode array detector. All solvents were of HPLC quality. Flash purification was carried out on a Biotage Selekt system. Microplate readings were taken on a BioTek Cytation 3 Cell Imaging Multi-Mode Reader.

### Collection Sites for Bacterial Isolation

Egg collars for isolation of bacterial strains were collected from three sites in SW Florida over multiple seasons—Dec. 2018, Dec. 2019, Feb. 2023, and Jan. 2024 (permit number: SAL-19-2113-SR; GPS coordinates: 26.591972, −82.068167; 26.681361, −82.089000; and 26.665222, −82.105278) and one site in W. Puerto Rico in Dec. 2022 (GPS coordinates: 17.978961, −67.213472). On the same day of collection, small portions of each egg collar were surface sterilized by submerging in 70% ethanol (*aq*) for 30 s, rinsed with sterile water (×3), and then ground up in sterile water by using a sterilized glass rod. Aliquots of this liquid were plated on various solid agar plates for isolation. Isolation media included marine agar, R2A in seawater, chitin in seawater, and RAM in seawater, all containing 50 mg/L of both nystatin and cylcoheximide (see Table S3). Isolation plates were incubated at room temp (rt) and monitored for bacterial growth. Colonies were subinoculated on yeast extract malt extract (YEME) and this process was repeated until pure cultures were obtained. All isolated bacterial strains were cryopreserved in 25% glycerol/water and identified to the genus level by using 16S rRNA techniques.

### Bacterial Chemical Fraction Library Generation

All bacterial strains were grown in 1 L of YEME, R2A and A-media in seawater (see Table S3) containing HP-20 (15 g/L), XAD4 (7.5 g/L) and XAD7 (7.5 g/L) resins to capture the produced metabolites. Cultures were grown for 7 d at 30 °C, shaking at 200 rpm. After 7 d, the resins from each media condition were combined, and the cultures were filtered through miracloth in order to recover the resins. The combined resin mixture was extracted with methanol (MeOH) and acetone for up to 16 h at rt. The organic extracts were combined and dried on 4 g of Celite to yield crude extracts. The crude extracts were semipurified with flash chromatography using 8 g of Phenomenex Supra C8 (50 μm, 65 Å) with a step-gradient −100% H_2_O (A - 40 mL), 15% MeOH/H_2_O (B - 40 mL), 30% MeOH/H_2_O (C - 40 mL), 45% MeOH/H_2_O (D - 40 mL), 60% MeOH/H_2_O (E - 40 mL), 80% MeOH/H_2_O (F - 40 mL) and 100% MeOH (G/H - 80 mL). Each eluate was transferred to a 40 mL vial and mixed thoroughly. An aliquot (3 mL) of each fraction was transferred to deep 48-well plates (VWR: 12000–728) with the fractions of each individual bacterial strain representing one column of the plate. Plates and vials were dried under vacuum. Vials were tared upon drying to completion and these weights were used to back calculate the amount of material in individual wells. Each fraction in the deep 48-well plates were resuspended at 10 or 50 mg/mL when not possible to resuspend at the lower concentration. Fractions were then transferred manually to 96-well plates. Fractions originally at 50 mg/mL were then diluted to 10 mg/mL at this point.

### Bacterial Strains and Biofilm Inhibition for Pathogens

*Pseudomonas aeruginosa* (*ΔmexABΔoprM*) and *Staphylococcus aureus* (ATCC BAA-2313) were used in both the biofilm disruption and inhibition assays. Overnight cultures of the strains were grown in Luria–Bertani (LB) medium (see Table S3). The biofilm inhibition and disruption assays were performed as previously described by Hancock et al.,^43^ except for the following variations. For the biofilm inhibition assay, 2 μL of each fraction (10 mg/mL in DMSO) was added to fresh 96-well plates in quadruplicate. Overnight cultures of each pathogen were diluted to a starting OD_600_ of 0.01 in M63 liquid broth (see Table S3) and 98 μL was transferred to each well, including solvent controls. Inoculated plates were incubated in a static incubator for 16 to 20 h at 37 °C. Bacterial growth was measured at 600 nm to ascertain whether the fractions exhibited antibacterial properties. Adhered biofilms were stained with 0.1% (wt/vol) crystal violet (*aq*) and placed on an orbital shaker at low speed for 30 min. Planktonic cells were washed with water, the plates were dried (30–60 min) at rt and then the crystal violet-bound biofilms were solubilized using 130 μL of 30% acetic acid (*aq*). The solubilized material was transferred to new 96-well plates and absorbances (595 nm) measured. Raw absorbance readings were normalized to a vehicle control (negative control) and active fractions were described as those that did not negatively impact bacterial growth but inhibited >50% of the biofilm formation. All active fractions were then prioritized for biofilm disruption assays.

### Biofilm Disruption Assay

In the biofilm disruption assay, *P. aeruginosa* and *S. aureus* biofilms were first established overnight and bacterial growth was measured at OD_600_ to ensure uniformity in growth across the plate. The spent media and planktonic bacteria were aspirated, and the plates were gently rinsed with fresh media three times. Fresh media (108 μL) was again added to the plates and 2 μL of each of the library fractions in DMSO (10 mg/mL) were added in quadruplicate. The plates were incubated under static conditions for 16 to 20 h at 37 °C. After incubation, OD_600_ was recorded to measure bacterial growth, spent supernatant media was discarded and the plates were rinsed (x3) with DI water and left for 30–60 min to air-dry at rt. The adhered biofilms were stained with 0.1% (wt/vol) crystal violet (*aq*) and placed on an orbital shaker at low speed for 30 min. Planktonic cells were washed, the plates were dried (30–60 min) at rt and then the crystal violet bound biofilms were solubilized in 30% acetic acid (*aq*). The solubilized material was transferred to new 96-well plates prior to recording the absorbances (595 nm). Active fractions were described as those that did not negatively impact bacterial growth but eradicated >50% of biofilm as measured by spectrophotometric measurements.

### Bioassay-Guided Isolation of Pseudochelin A

*P. piscicida* EM138, one of the producing strains, was grown on a large scale (8 × 1 L) in R2A broth [(−) agar] medium containing 30 g/L of resins (2:1:1 HP20/XAD4/XAD7) for 12 d at 30 °C, 200 rpm. Resins were filtered using miracloth and extracted with acetone (4 h, rt) and MeOH (16 h, rt). The organic material was filtered through a coffee filter, 8 g of Celite was added, and the material was dried under vacuum. The dried crude extract was fractionated on a Biotage Flash Purification System following a stepwise gradient [100% H_2_O (fraction A), 75% H_2_O/MeCN (fraction B), 50% H_2_O/MeCN (fraction C), 25% H_2_O/MeCN (fraction D) and 100% MeCN (fractions E and F)] using 40 mL of each eluent. Fraction C (358 mg) contained the targeted compound and was thus chosen for further purification by HPLC. HPLC equipped with a Phenomenex Luna 5 μm Phenyl-Hexyl C18 (250 × 10 mm) column with a flow rate of 3 mL/min, hold at 10% MeCN + 0.1% formic acid (FA)/H_2_O + 0.1% FA for 5 min then gradient to 25% MeCN + 0.1% FA/75% H_2_O + 0.1% FA over 35 min yielded pseudochelin A (**1**) eluting between 30 to 33 min.

### Dose-Dependent Biofilm Inhibition Assay

Pseudochelin A was dissolved in DMSO to a stock concentration of 10 mg/mL. The stock solution was 2-fold serially diluted to yield 12 stock concentrations (10–0.0049 mg/mL). An aliquot (2 μL) of each concentration was used in the assay following the exact protocol described above in the section “Bacterial Strains and Biofilm Inhibition for Pathogens”.

### Bacterial Strains and Biofilm Inhibition of Marine Bacteria

Five bacterial strains, representing three classes [two *Bacillus* sp. (Bacilli), one *Labrenzia* sp. (Alphaproteobacteria), and two *Isoptericola* sp. (Actinobacteria)], were isolated from moon snail egg collars at the same time as *P. piscicida* EM138 (pseudochelin A producing strain) and produced biofilms in the laboratory. Therefore, these strains were used to evaluate the potential of compound **1** to inhibit biofilm formation in environmental isolates. The biofilm inhibition assay was performed as described above except for the following variations. The environmental isolates were diluted to a starting OD_600_ of 0.01 in M63 liquid broth prepared with artificial seawater and 98 μL was transferred to each well, including the vehicle control. Inoculated plates were incubated in a static incubator for 40 to 48 h at 30 °C and residual biofilms were quantified following the same methods described above.

### Biofilm Inhibition Assay against Marine Bacteria

Pseudochelin A was dissolved in DMSO to stock concentrations of 5 and 1.7 mg/mL. The stock solutions were diluted to final concentrations of 100 and 34 μg/mL, respectively, by mixing 2 μL of each solution with 98 μL of M63 liquid broth with seawater in 96-well plates in triplicate. Overnight cultures of the strains were grown in LB medium with artificial seawater, diluted to a starting OD_600_ of 0.01 in M63 liquid broth with seawater, and 98 μL was transferred to each well, including solvent controls. Inoculated plates were incubated in a static incubator for 40 to 48 h at 30 °C. Quantification of biofilms was performed as described above in the section “Bacterial Strains and Biofilm Inhibition for Pathogens”.

### Dose-Dependent Biofilm Inhibition Assay with Ferric Chloride

Ferric chloride (FeCl_3_) was dissolved in water to a stock concentration of 10 mM. The stock solution was 2-fold serially diluted to yield 10 stock concentrations (10–0.019 mM). An aliquot (2 μL) of each concentration was used in the assay following the protocol described above in the “Bacterial Strains and Biofilm Inhibition for Pathogens” section.

### Biofilm Inhibition Assay with Iron Supplementation

A 10 mM stock concentration of FeCl_3_ was serially diluted by 2-fold to yield three concentrations: 0.625, 0.313, and 0.156 mM. Pseudochelin A was dissolved in DMSO at a concentration of 4.4 mM. In each well, 2 μL of pseudochelin A, 2 μL of FeCl_3_, and 96 μL of M63 liquid broth were mixed. The rest of the assay followed the protocol described above in the “Bacterial Strains and Biofilm Inhibition for Pathogens” section.

### Mass Spectrometry Metabolomics of Biofilm Inhibition Fractions

The thirty-nine fractions that inhibited *S. aureus* biofilms were resuspended in 50% MeOH/H_2_O at a concentration of 1 mg/mL and analyzed by LR-LCMS for the presence of pseudochelin A. The LC was equipped with a Phenomenex Kinetex 5 μm C8 (100 × 3.0 mm) column and run under the following method: flow rate of 0.3 mL/min, hold 10% MeCN/H_2_O + 0.1% formic acid (FA) for 3 min then gradient to 100% MeCN + 0.1% formic acid over 14 min. Pseudochelin A eluted between 8 to 9 min and was present in 25 fractions.

### Mass Spectrometry Metabolomics on the Fraction Library

Upon thawing fractions A–H (10 mg/mL in DMSO), 2 μL of fractions A–D and E–H were combined in an LCMS vial containing 90 μL of 50% MeCN/H_2_O. An aliquot (2 μL) of this mixture was analyzed by LC-q-ToF under the following conditions: Phenomenex Kinetex 3 μm Evo C18 column (2.6 × 100 mm), 0.3 mL/min, hold 30% MeCN + 0.1% FA/70% H_2_O + 0.1% FA for 1 min then gradient to 100% MeCN + 0.1% FA over 12.5 min and held for 1 min before re-equilibration at 30% MeCN + 0.1% FA/70% H_2_O + 0.1% FA for 4 min. MS data were collected using an Agilent 6530 q-ToF in positive mode using Agilent MassHunter software. MS1 data were collected from 100 to 2,000 *m*/*z*. MS/MS spectra were collected using data-dependent acquisition, for which the top three abundant ions in the MS1 scan were selected for fragmentation; dynamic exclusion was employed to avoid fragmenting the same ion more than twice in a 2 min range. In addition, an exclusion list was utilized that contained features that were present in the control extract of the three-nutrient media (YEME, R2A and A-media). Collision-induced dissociation was applied using a linear formula that applied a higher voltage for larger molecules [CID voltage = 10 + 0.02*(*m*/*z*)]. MS scan rate 1/s, MS/MS scan rate 3/s, source gas temperature 330 °C, gas flow 34 L/min, nebulizer 35 psig. All LC-MS/MS data collected using the Agilent on the bacterial fractions are publicly available: https://gnps.ucsd.edu/ProteoSAFe/status.jsp?task=8724e892b45f4db091944806b4d25cce.

### Global Natural Product Social (GNPS) Molecular Networking on Fraction Library

The raw MS files were converted into mgf by MSConvert and analyzed on the GNPS platform. A molecular network was created with the online workflow (https://ccms-ucsd.github.io/GNPSDocumentation/) on the GNPS Web site (http://gnps.ucsd.edu).^40^ The data were filtered by removing all MS/MS fragment ions within ±17 Da of the precursor *m*/*z*. MS/MS spectra were window filtered by choosing only the top 6 fragment ions in the ±50 Da window throughout the spectrum. The precursor ion mass tolerance was set to 1 Da and a MS/MS fragment ion tolerance to 0.5 Da. A network was then created where edges were filtered to have a cosine score above 0.65 and more than 6 matched peaks. Further, edges between two nodes were kept in the network, if and only if, each of the nodes appeared in each other’s respective top 10 most similar nodes. Finally, the maximum size of a molecular family was set to 100, and the lowest scoring edges were removed from molecular families until the molecular family size was below this threshold. The spectra in the network were then searched against GNPS’ spectral libraries. The library spectra were filtered in the same manner as the input data. All matches kept between network spectra and library spectra were required to have a score above 0.7 and at least 6 matched peaks.

### Genome Sequencing of *P. piscicida* EM138 and AntiSMASH Analysis

*P. piscicida* EM138 was grown in LB media to OD_600_ of 2 (∼10^9^ cells). Cells were harvested by centrifugation, decanted, frozen at −20 °C, and shipped on ice to SeqCenter (Pittsburgh, PA) for genomic DNA extraction. DNA was sequenced using the hybrid sequencing option (600 Mbp Nanopore sequencing). Sequencing, assembly, and annotation was performed by SeqCenter (Table S5). Briefly, Porechop^44^ was used to trim residual adapter sequences from the Oxford Nanopore Technology (ONT) reads that may have been missed during base-calling and demultiplexing. *De novo* genome assemblies were generated from the ONT read data with Flye^45^ under the nanohq (ONT high-quality reads) model. Additional Flye options initiated the assembly by first using reads longer than an estimated N50 based on a genome size of 6Mbp. Assembled contigs were evaluated for circularization via Circulator.^46^ Assembly annotation was then performed with Bakta^47^ using the Bakta v5 database. Finally, assembly statistics were recorded with QUAST.^48^ The assembled genome was deposited into the NCBI genome database (accession number PRJNA1038720). The genome was run in a BLAST search against NCBI databases and was identified as a *P. piscicida* EM138. The genome of *P. piscicida* EM138 was analyzed with AntiSMASH (v. 7.0.1). Fifteen BGC’s were identified, including a pseudochelin A gene cluster.

### Single Egg Collar DNA Extraction

Single egg collars (10) were collected and preserved in denatured alcohol during the Feb. 2023 field excursion in SW Florida at two distinct sites (26.681361, −82.089000; 26.665222, −82.105278). Samples were stored at 4 °C until ready for use. A small section (500–800 mg) of the egg collar was weighed, and DNA was extracted with the NucleoSpin Soil DNA Isolation Kit (Takara) as instructed. Extracted DNA (eDNA) concentration and quality was measured on a NanoDrop 2000 (ThermoFisher), and stored at −20 °C until it was used.

### PCR Screening of Egg Mass Collars Environmental DNA

Primers were designed to amplify NRPS-A from the eDNA (For: 5′-GACAGCTATTAGCTATGAGCGGTTAGTACAGCAAG-3′; Rev: 5′-GTAACACAGTGTATCACCGCGGCTTCAAGAATG-3′). DNA was amplified with NEBNext High-Fidelity 2X PCR Master Mix (New England BioLabs, Inc.) in a BioRad C1000 Touch Thermal Cycler (denaturing at 95 °C, 30 s, annealing at 55 °C, 30 s, extension 1 min at 72 °C, repeat 45×). DNA was visualized with SYBR Safe DNA Gel Stain (ThermoFisher). Using this PCR method, the presence of the pseudochelin A NRPS-A gene in the eDNA described above was detected in three of ten samples. To confirm that the PCR product represented the targeted NRPS-A, the band from one sample was gel purified, reamplified, and sequenced through the Illumina platform. The resulting paired-end sequences were imported into QIIME2 (v.2020.6) for demultiplexing, denoising and removal of chimera sequences. Quality filters “--p-trim-left-f”, “--p-trim-left-r”, “--p-max-ee-f”, “--p-max-ee-r” and “--p-chimera-method” were set to 10, 10, 1.0, 1.0 and “consensus”, respectively. Any sequences <300 bp and containing a stop codon in all three reading frames were manually excluded. This yielded three unique sequences, all from *P. piscicida*, including a match to NRPS-A (Table S4).

## Data Availability

The original NMR data (FIDs) of compound **1** is available via NP-MRD (accession #: NP0332751) at https://np-mrd.org/. The MS data used for the GNPS networking is available via MassIVE (accession #: MSV000094443) at https://massive.ucsd.edu/ProteoSAFe/static/massive.jsp.

## Supporting Information Available

All media recipes, analytical data for pseudochelin A (NMR, MS, and ECD), additional biological assays, details on bacterial fraction library, and sequencing data (PDF)
