# Supplementary material for: Discovery of Biofilm Inhibitors from the Microbiota of Marine Egg Masses
Source: J Nat Prod. 2024 May 30;87(6):1635–42. doi: 10.1021/acs.jnatprod.4c00376 (PMC11217947; doi:10.1021/acs.jnatprod.4c00376)
Supplement: Supplementary file 1 — np4c00376_si_001.pdf [file np4c00376_si_001.pdf]

Discovery of Biofilm Inhibitors from the Microbiota of Marine Egg Masses

Lois Kyei,[†] Karla Piedl,[†] Carla Menegatti,[†] Eleanor M. Miller,[†] Emily Mevers[†,]*

[†]Department of Chemistry, Virginia Tech, Blacksburg, Virginia, USA

Table of Contents

Figure S1: ^{1}H NMR (600 MHz) of pseudochelin A (**1**) in $(CD_3)_2CO$……………………………….3
Figure S2: HSQC spectrum of pseudochelin A (**1**) in $(CD_3)_2CO$……………………………….3
Figure S3: COSY spectrum of pseudochelin A (**1**) in $(CD_3)_2CO$……………………………….4
Figure S4: ECD spectrum of pseudochelin A (**1**) in MeOH confirming *S* configuration…………4
Figure S5: HRESIMS spectrum of pseudochelin A (**1**)……………………………………………5
Figure S6: LR-MS/MS (ESI) fragmentation data of pseudochelin A (**1**)…………………………5
Figure S7: Dose dependency assays of pseudochelin (**1**) against both *S. aureus* and *Isoptericola* sp, with the x-axis in μM………………………………………………………………5
Figure S8: Biofilm disruption assay of pseudochelin A (**1**)……………………………………..6
Figure S9: Inhibition of biofilm formation by pseudochelin A (**1**) of ecologically relevant strains…………………………………………………………………………………………………6
Figure S10: Addition of exogenous iron ($FeCl_3$) in the presence of pseudochelin A (**1**) abolishes biofilm inhibition activity against *S. aureus*……………………………………………………7
Figure S11: Addition of exogenous iron ($FeCl_3$) inhibits biofilm formation in *S. aureus* in a dose-dependent manner up to concentrations below 12.5 μM……………………………………………7
Table S1: Bacterial strains that compose the fraction library……………………………………8
Table S2: Strains and precursor masses of nodes in the GNPS cluster containing 136 Da neutral loss……………………………………………………………………………………………31
Table S3: Media Recipes………………………………………………………………………32
Figure S10: Fragmentation of precursor mass 386.037 m/z…………………………………..33
Figure S11: Fragmentation of precursor mass 386.172 m/z…………………………………...33
Figure S12: Fragmentation of precursor mass 395.159 m/z……………………………………33
Figure S13: Fragmentation of precursor mass 400.211 m/z…………………………………...34
Figure S14: Fragmentation of precursor mass 422.133 m/z…………………………………...34
Figure S15: Fragmentation of precursor mass 427.149 m/z…………………………………...34
Figure S16: Fragmentation of precursor mass 446.139 m/z…………………………………...35
Figure S17: Fragmentation of precursor mass 464.151 m/z……………………………………35
Figure S18: Fragmentation of precursor mass 495.224 m/z…………………………………...35
Figure S19: Fragmentation of precursor mass 509.240 m/z…………………………………...36
Figure S20: Fragmentation of precursor mass 520.174 m/z……………………………………36
Figure S21: Biofilm inhibition by moon snail egg mass collars chemical fractions collected from Florida and Puerto Rico…………………………………………………………………………...37
Figure S22: PCR amplification of NRPS adenylation domain ……………………………….37
Figure S23: PCR amplification of NRPS-A domain for sequencing……………………………38
Table S4: Protein matches for sequences obtained from Illumina sequencing…………………39
Table S5: Genome assembly and annotation parameters…………………………………………40

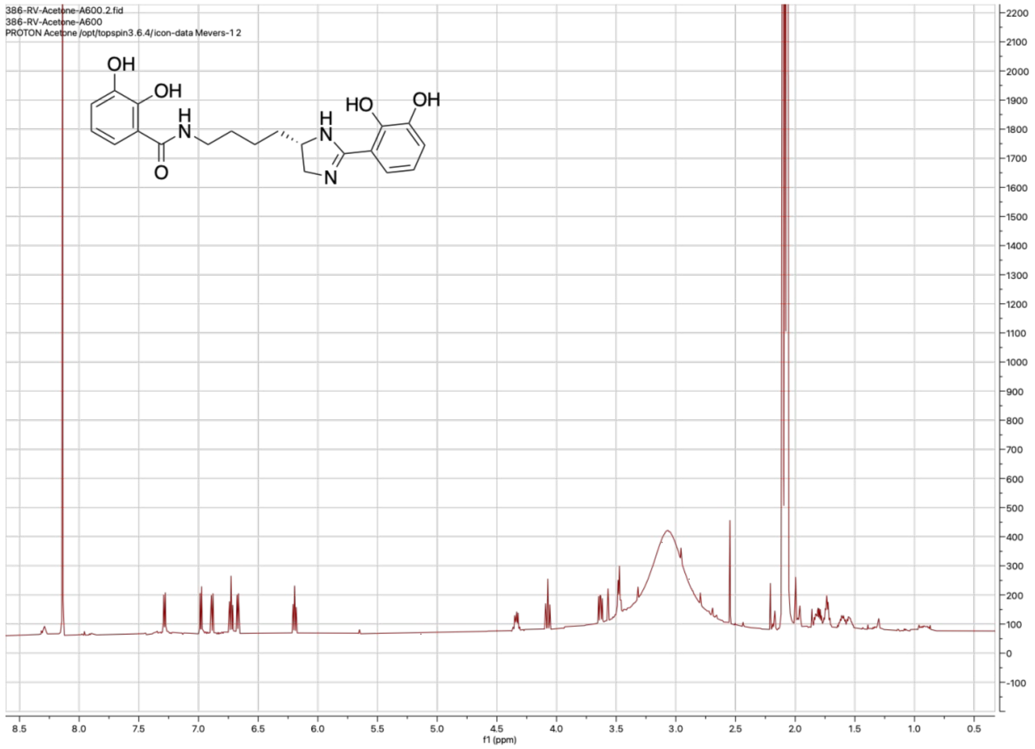

Figure S1: ^{1}H NMR (600 MHz) of pseudochelin A (**1**) in $(CD_3)_2CO$.

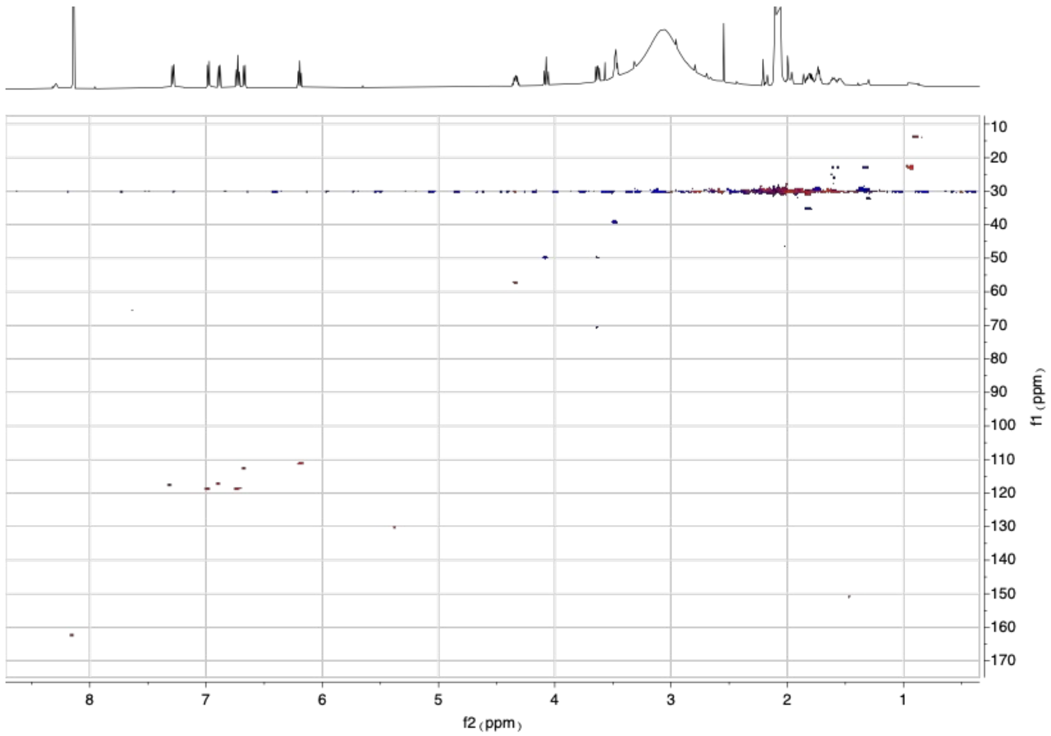

Figure S2: gHSQC spectrum of pseudochelin A (**1**) in $(CD_3)_2CO$.

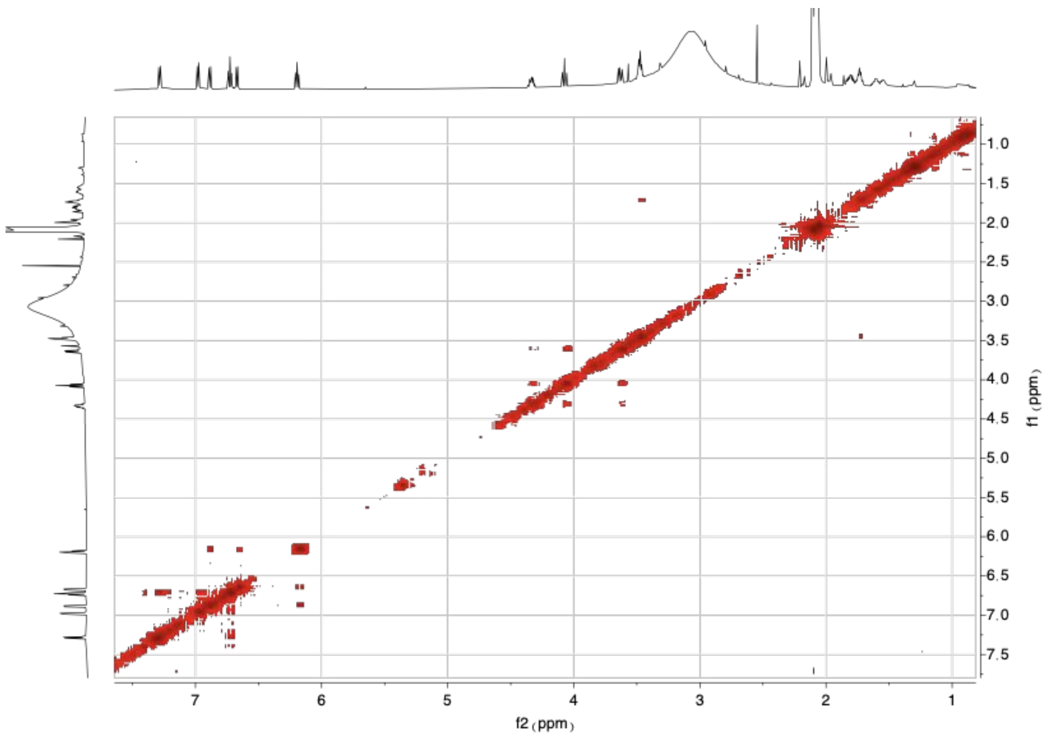

Figure S3: dqfCOSY spectrum of pseudochelin A (**1**) in $(CD_3)_2CO$.

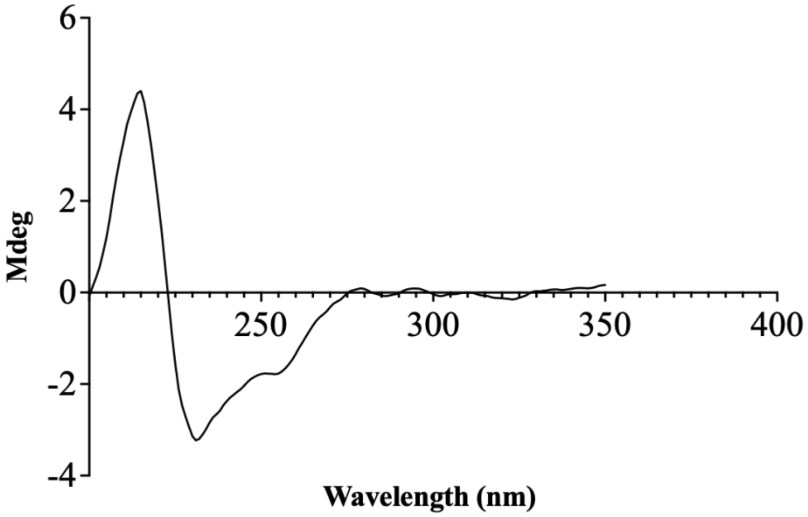

Figure S4: ECD spectrum of pseudochelin A (**1**) in MeOH confirming *S* configuration

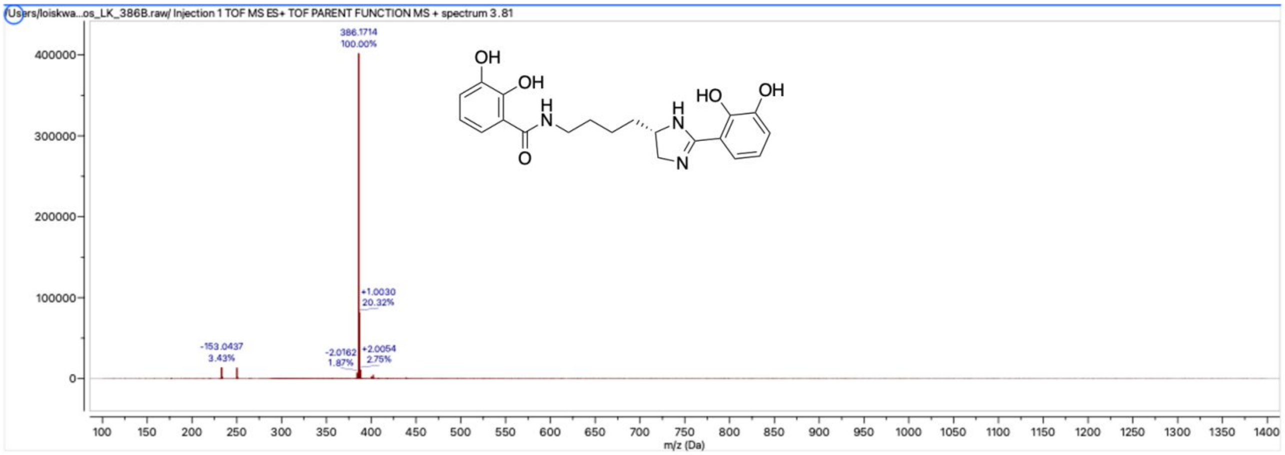

Figure S5: HRESIMS (q-ToF) spectrum of pseudochelin A (**1**)

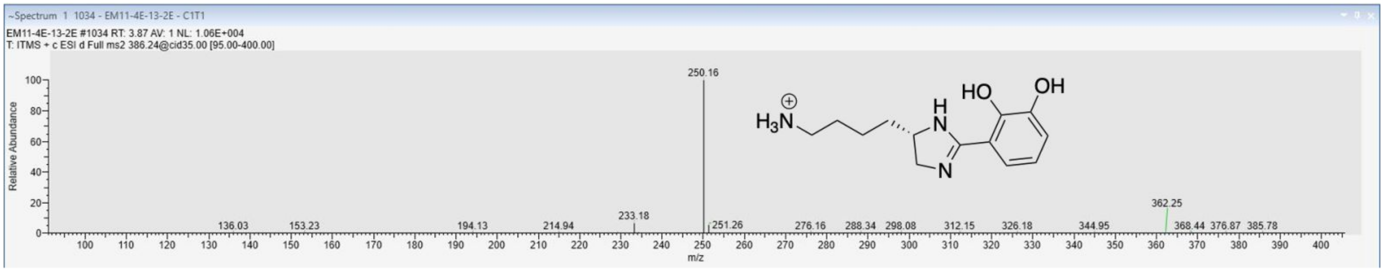

Figure S6: LR-MS/MS (ESI LTQ) fragmentation data of pseudochelin A (**1**)

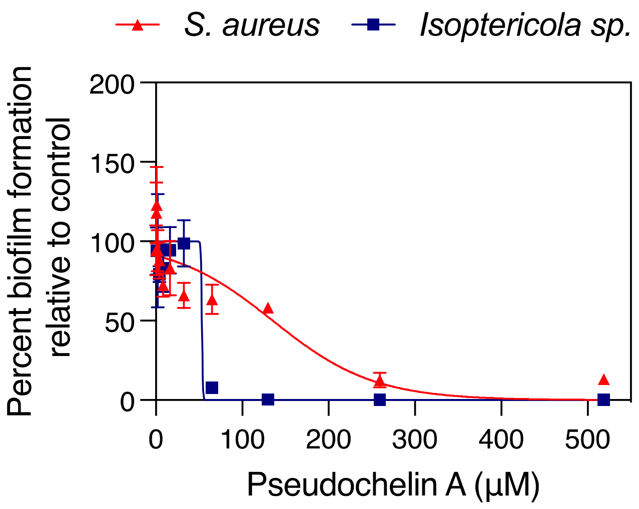

Figure S7: Dose dependency assays of pseudochelin (**1**) against both *S. aureus* and *Isoptericola* sp, with the x-axis in µM.

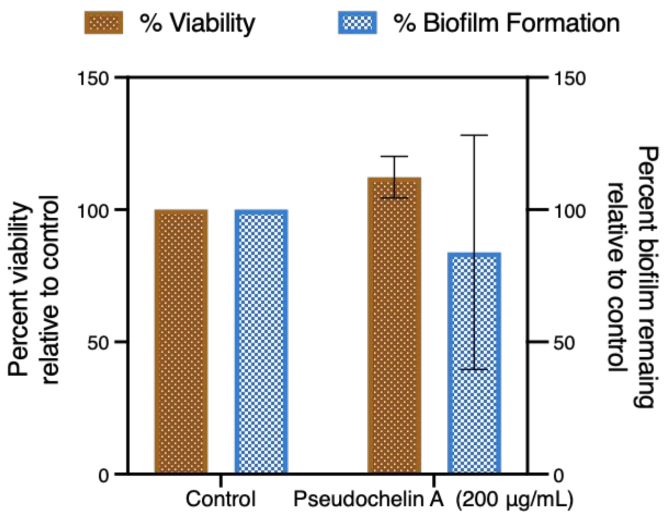

Figure S8: Biofilm disruption assay of pseudochelin A (**1**). Biofilms were preformed prior to treatment with **1**. Compound **1** had no impact on disrupting preformed biofilms.

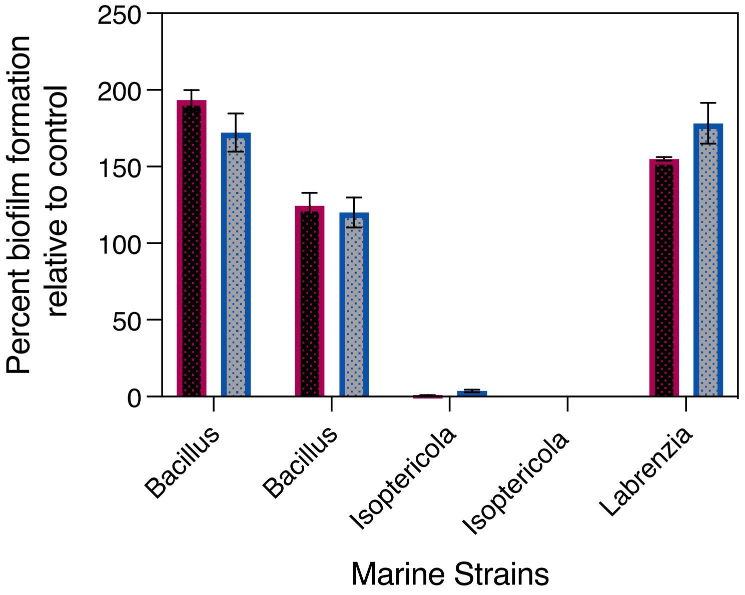

Figure S9: Inhibition of biofilm formation by pseudochelin A (**1**) of ecologically relevant strains. Compound **1** was tested at two concentration, 259 (red) and 88.2 (blue) µM, against five marine strains isolated at the same time as *Pseudoalteromonas piscicida* EM138.

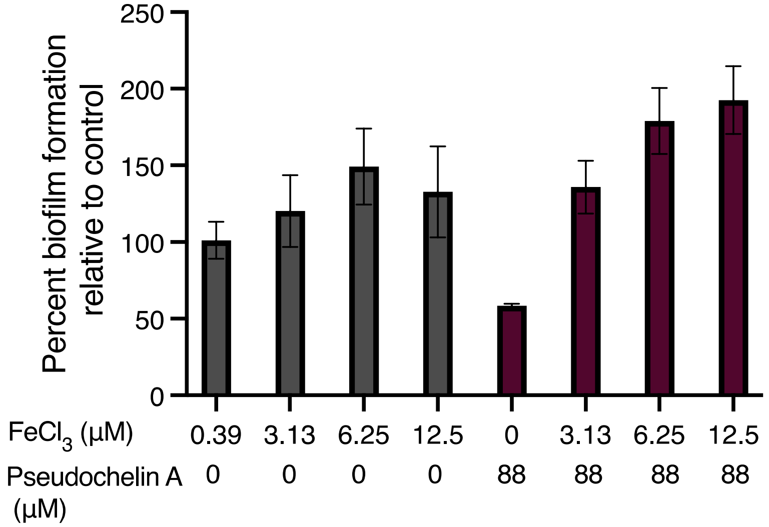

Figure S10: Addition of exogenous iron ($FeCl_3$) in the presence of pseudochelin A (**1**) abolishes biofilm inhibition activity against *S. aureus*.

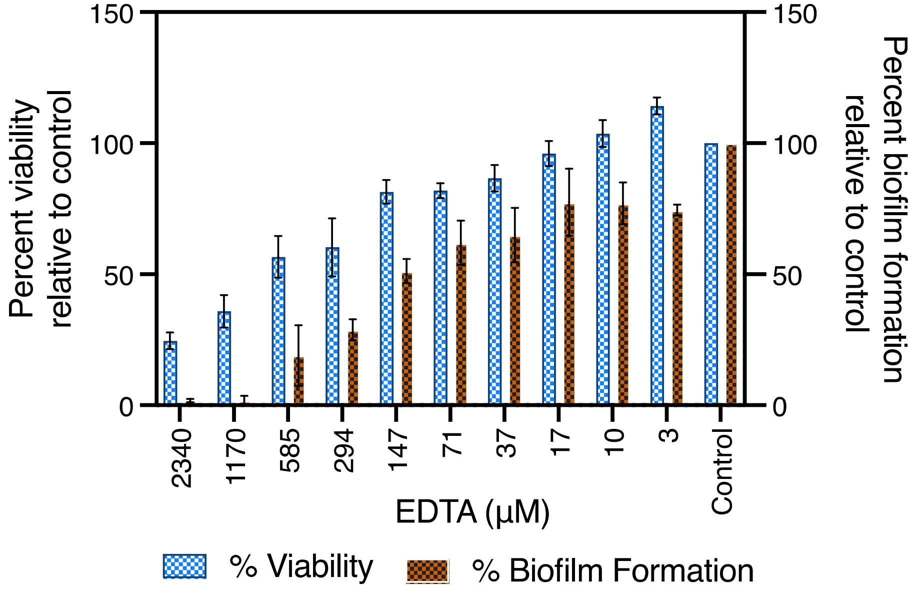

Figure S11: Addition of exogenous iron ($FeCl_3$) inhibits biofilm formation in *S. aureus* in a dose-dependent manner up to concentrations below 12.5 µM.

Table S1: Bacterial strains that compose the fraction library					
**Strain**	**Fraction code**	**Source**	**Biofilm Inhibition**	**Biofilm Disruption**	**Pseudochelin A in extract**
Photobacterium sp. (EM1-1)	EM1-1A	FL Moon snail	No	No	
	EM1-1B		No	No	
	EM1-1C		No	No	
	EM1-1D		No	No	
	EM1-1E		No	No	
	EM1-1F		No	No	
	EM1-1G		No	No	
	EM1-1H		No	No	
Fictibacillus sp. (EM1-2)	EM1-2A	FL Moon snail	No	No	
	EM1-2B		No	No	
	EM1-2C		No	No	
	EM1-2D		No	No	
	EM1-2E		No	No	
	EM1-2F		No	No	
	EM1-2G		No	No	
	EM1-2H		No	No	
Pseudoalteromonas sp. (EM1-3)	EM1-3A	FL Moon snail	No	No	
	EM1-3B		No	No	
	EM1-3C		No	No	
	EM1-3D		No	No	
	EM1-3E		No	No	
	EM1-3F		No	No	
	EM1-3G		No	No	
	EM1-3H		No	No	
Pseudoalteromonas sp. (EM1-4)	EM1-4A	FL Moon snail	No	No	
	EM1-4B		No	No	
	EM1-4C		No	No	
	EM1-4D		No	No	
	EM1-4E		No	No	
	EM1-4F		No	No	
	EM1-4G		No	No	
	EM1-4H		No	No	
Vibrio sp. (EM1-5)	EM1-5A	FL Moon Snail	Yes (*P. aeruginosa*)	No	
	EM1-5B		Yes (*P. aeruginosa*)	No	
	EM1-5C		No	No	

	EM1-5D		No	No	
	EM1-5E		No	No	
	EM1-5F		No	No	
	EM1-5G		No	No	
	EM1-5H		No	No	
Photobacterium sp. (EM1-6)	EM1-6A	FL Moon Snail	No	No	
	EM1-6B		No	No	
	EM1-6C		No	No	
	EM1-6D		No	No	
	EM1-6E		No	No	
	EM1-6F		No	No	
	EM1-6G		No	No	
	EM1-6H		No	No	
Vibrio sp. (EM2-1)	EM2-1A	FL Moon Snail	No	No	
	EM2-1B		No	No	
	EM2-1C		No	No	
	EM2-1D		No	No	
	EM2-1E		No	No	
	EM2-1F		No	No	
	EM2-1G		No	No	
	EM2-1H		No	No	
Photobacterium sp. (EM2-2)	EM2-2A	FL Moon Snail	No	No	
	EM2-2B		No	No	
	EM2-2C		No	No	
	EM2-2D		No	No	
	EM2-2E		Yes (*P. aeruginosa*)	No	
	EM2-2F		No	No	
	EM2-2G		No	No	
	EM2-2H		No	No	
Pseudoalteromonas sp. (EM2-3)	EM2-3A	FL Moon Snail	No	No	
	EM2-3B		No	No	
	EM2-3C		No	No	
	EM2-3D		No	No	
	EM2-3E		No	No	
	EM2-3F		No	No	
	EM2-3G		No	No	
	EM2-3H		No	No	
Vibrio sp. (EM2-4))	EM2-4A	FL Moon Snail	No	No	
	EM2-4B		No	No	

	EM2-4C		No	No	
	EM2-4D		Yes (*P. aeruginosa*)	No	
	EM2-4E		Yes (*P. aeruginosa*)	No	
	EM2-4F		No	No	
	EM2-4G		No	No	
	EM2-4H		No	No	
Bacillus sp. (EM2-5)	EM2-5A	FL Moon Snail	No	No	
	EM2-5B		No	No	
	EM2-5C		No	No	
	EM2-5D		No	No	
	EM2-5E		Yes (*P. aeruginosa*)	No	
	EM2-5F		No	No	
	EM2-5G		No	No	
	EM2-5H		No	No	
Pseudoalteromonas sp. (EM3-1)	EM3-1A	FL Moon Snail	No	No	
	EM3-1B		No	No	
	EM3-1C		No	No	
	EM3-1D		No	No	
	EM3-1E		Yes (*P. aeruginosa*)	No	
	EM3-1F		No	No	
	EM3-1G		No	No	
	EM3-1H		No	No	
Pseudoalteromonas sp. (EM3-2)	EM3-2A	FL Moon Snail	No	No	
	EM3-2B		No	No	
	EM3-2C		No	No	
	EM3-2D		No	No	
	EM3-2E		No	No	
	EM3-2F		No	No	
	EM3-2G		No	No	
	EM3-2H		No	No	
Vibrio sp. (EM3-3)	EM3-3A	FL Moon Snail	No	No	
	EM3-3B		No	No	
	EM3-3C		No	No	
	EM3-3D		No	No	
	EM3-3E		Yes (*P. aeruginosa*)	No	

	EM3-3F		No	No	
	EM3-3G		No	No	
	EM3-3H		No	No	
Vibrio sp. (EM3-4)	EM3-4A	FL Moon Snail	No	No	
	EM3-4B		No	No	
	EM3-4C		No	No	
	EM3-4D		No	No	
	EM3-4E		No	No	
	EM3-4F		No	No	
	EM3-4G		No	No	
	EM3-4H		No	No	
Vibrio sp. (EM3-5)	EM3-5A	FL Moon Snail	No	No	
	EM3-5B		No	No	
	EM3-5C		No	No	
	EM3-5D		No	No	
	EM3-5E		Yes (*P. aeruginosa*)	No	
	EM3-5F		No	No	
	EM3-5G		No	No	
	EM3-5H		No	No	
Bacillus sp. (EM3-6)	EM3-6A	FL Moon Snail	No	No	
	EM3-6B		No	No	
	EM3-6C		No	No	
	EM3-6D		No	No	
	EM3-6E		No	No	
	EM3-6F		No	No	
	EM3-6G		No	No	
	EM3-6H		No	No	
Vibrio sp. (EM4-1)	EM4-1A	FL Moon Snail	No	No	
	EM4-1B		No	No	
	EM4-1C		Yes (*P. aeruginosa*)	No	
	EM4-1D		No	No	
	EM4-1E		No	No	
	EM4-1F		No	No	
	EM4-1G		No	No	
	EM4-1H		No	No	
Vibrio sp. (EM4-2)	EM4-2A	FL Moon Snail	No	No	
	EM4-2B		No	No	
	EM4-2C		No	No	

	EM4-2D		No	No	
	EM4-2E		No	No	
	EM4-2F		No	No	
	EM4-2G		No	No	
	EM4-2H		No	No	
Marinomonas sp. (EM4-3)	EM4-3A	FL Moon Snail	No	No	
	EM4-3B		No	No	
	EM4-3C		No	No	
	EM4-3D		No	No	
	EM4-3E		Yes (*P. aeruginosa*)	No	
	EM4-3F		No	No	
	EM4-3G		No	No	
	EM4-3H		No	No	
Bacillus sp. (EM4-4)	EM4-4A	FL Moon Snail	No	No	
	EM4-4B		No	No	
	EM4-4C		No	No	
	EM4-4D		No	No	
	EM4-4E		Yes (*P. aeruginosa*)	No	
	EM4-4F		No	No	
	EM4-4G		No	No	
	EM4-4H		No	No	
Bacillus sp. (EM2-6)	EM2-6A	FL Moon Snail	No	No	
	EM2-6B		No	No	
	EM2-6C		No	No	
	EM2-6D		No	No	
	EM2-6E		Yes (*P. aeruginosa*)	No	
	EM2-6F		No	No	
	EM2-6G		No	No	
	EM2-6H		No	No	
Vibrio sp. (EM5-1)	EM5-1A	FL Moon Snail	No	No	
	EM5-1B		No	No	
	EM5-1C		No	No	
	EM5-1D		No	No	
	EM5-1E		No	No	
	EM5-1F		No	No	
	EM5-1G		No	No	
	EM5-1H		No	No	

Pseudoalteromonas sp. (EM5-2)	EM5-2A	FL Moon Snail	No	No	
	EM5-2B		No	No	
	EM5-2C		No	No	
	EM5-2D		No	No	
	EM5-2E		No	No	
	EM5-2F		No	No	
	EM5-2G		No	No	
	EM5-2H		No	No	
Bacillus sp. (EM5-3)	EM5-3A	FL Moon Snail	No	No	
	EM5-3B		No	No	
	EM5-3C		No	No	
	EM5-3D		No	No	
	EM5-3E		No	No	
	EM5-3F		Yes (*P. aeruginosa*)	No	
	EM5-3G		No	No	
	EM5-3H		No	No	
Bacillus sp. (EM5-4)	EM5-4A	FL Moon Snail	No	No	
	EM5-4B		No	No	
	EM5-4C		No	No	
	EM5-4D		No	No	
	EM5-4E		No	No	
	EM5-4F		Yes (*P. aeruginosa*)	No	
	EM5-4G		No	No	
	EM5-4H		No	No	
Curtobacterium sp. (EM6-1)	EM6-1A	Sergeant Major Eggs (New England Aquarium)	No	No	
	EM6-1B		Yes (*S. aureus*)	Yes (*S. aureus*)	No
	EM6-1C		No	No	
	EM6-1D		No	No	
	EM6-1E		No	No	
	EM6-1F		Yes (*P. aeruginosa*)	No	
	EM6-1G		No	No	
	EM6-1H		Yes (*S. aureus*)	No	No
Brachybacterium sp. (EM6-2)	EM6-2A	FL Gastropod Egg Casing	No	No	
	EM6-2B		Yes (*S. aureus*)	Yes (*S. aureus*)	No
	EM6-2C		No	No	
	EM6-2D		No	No	

	EM6-2E		No	No	
	EM6-2F		Yes (*P. aeruginosa*)	No	
	EM6-2G		No	No	
	EM6-2H		No	No	
Rothia sp. (EM6-3)	EM6-3A	Banded tulip egg casing (FL)	No	No	
	EM6-3B		No	No	
	EM6-3C		No	No	
	EM6-3D		Yes (*P. aeruginosa*)	No	
	EM6-3E		No	No	
	EM6-3F		Yes (*P. aeruginosa*)	No	
	EM6-3G		No	No	
	EM6-3H		Yes (*P. aeruginosa*)	No	
Brachybacterium sp. (EM6-4)	EM6-4A	Banded tulip egg casing (FL)	No	No	
	EM6-4B		No	No	
	EM6-4C		No	No	
	EM6-4D		No	No	
	EM6-4E		No	No	
	EM6-4F		No	No	
	EM6-4G		Yes (*S. aureus*)	No	No
	EM6-4H		No	No	
Tsukamurella sp. (EM6-5)	EM6-5A	FL Gastropod Egg Casing	No	No	
	EM6-5B		No	No	
	EM6-5C		No	No	
	EM6-5D		No	No	
	EM6-5E		No	No	
	EM6-5F		No	No	
	EM6-5G		No	No	
	EM6-5H		Yes (*P. aeruginosa*)	No	
Dietzia sp. (EM6-6)	EM6-6A	FL Gastropod Egg Casing	No	No	
	EM6-6B		No	No	
	EM6-6C		No	No	
	EM6-6D		No	No	
	EM6-6E		No	No	
	EM6-6F		No	No	
	EM6-6G		No	No	
	EM6-6H		Yes (*S. aureus*)	No	Yes

MEDIA	EM7-1A	Negative Control	No	No	
	EM7-1B		No	No	
	EM7-1C		No	No	
	EM7-1D		No	No	
	EM7-1E		No	No	
	EM7-1F		No	No	
	EM7-1G		No	No	
	EM7-1H		No	No	
Microbacterium sp. (EM7-2)	EM7-2A	FL Moon Snail	No	No	
	EM7-2B		No	No	
	EM7-2C		No	No	
	EM7-2D		No	No	
	EM7-2E		No	No	
	EM7-2F		No	No	
	EM7-2G		No	No	
	EM7-2H		No	No	
Arenibacter sp. (EM7-3)	EM7-3A	FL Moon Snail	No	No	
	EM7-3B		No	No	
	EM7-3C		Yes (*S. aureus*)	No	No
	EM7-3D		No	No	
	EM7-3E		No	No	
	EM7-3F		No	No	
	EM7-3G		No	No	
	EM7-3H		No	No	
Phaeobacter sp. (EM7-4)	EM7-4A	FL Moon Snail	No	No	
	EM7-4B		No	No	
	EM7-4C		No	No	
	EM7-4D		No	No	
	EM7-4E		No	No	
	EM7-4F		No	No	
	EM7-4G		No	No	
	EM7-4H		No	No	
Labrenzia sp. (EM7-5)	EM7-5A	FL Moon Snail	No	No	
	EM7-5B		No	No	
	EM7-5C		No	No	
	EM7-5D		No	No	
	EM7-5E		No	No	
	EM7-5F		No	No	
	EM7-5G		No	No	
	EM7-5H		No	No	
Labrenzia sp.	EM7-6A		No	No	

(EM7-6)	EM7-6B	FL Moon Snail	No	No	
	EM7-6C		No	No	
	EM7-6D		No	No	
	EM7-6E		Yes (*S. aureus*)	No	Yes
	EM7-6F		No	No	
	EM7-6G		No	No	
	EM7-6H		No	No	
Cobetia sp. (EM8-1)	EM8-1A	FL Moon Snail	No	No	
	EM8-1B		No	No	
	EM8-1C		No	No	
	EM8-1D		No	No	
	EM8-1E		No	No	
	EM8-1F		No	No	
	EM8-1G		No	No	
	EM8-1H		No	No	
Isoptericola sp. (EM8-2)	EM8-2A	FL Moon Snail	No	No	
	EM8-2B		No	No	
	EM8-2C		No	No	
	EM8-2D		No	No	
	EM8-2E		No	No	
	EM8-2F		No	No	
	EM8-2G		No	No	
	EM8-2H		No	No	
Streptomyces sp. (EM8-3)	EM8-3A	FL Moon Snail	No	No	
	EM8-3B		No	No	
	EM8-3C		No	No	
	EM8-3D		No	No	
	EM8-3E		No	No	
	EM8-3F		No	No	
	EM8-3G		No	No	
	EM8-3H		No	No	
Sanguibacter sp. (EM8-4)	EM8-4A	Sergeant Major Eggs (New England Aquarium)	No	No	
	EM8-4B		No	No	
	EM8-4C		No	No	
	EM8-4D		No	No	
	EM8-4E		No	No	
	EM8-4F		No	No	
	EM8-4G		No	No	
	EM8-4H		No	No	
Phaeobacter sp. (EM8-5)	EM8-5A	Banded tulip egg	No	No	
	EM8-5B		No	No	

	EM8-5C	casing (FL)	No	No	
	EM8-5D		No	No	
	EM8-5E		No	No	
	EM8-5F		No	No	
	EM8-5G		No	No	
	EM8-5H		No	No	
Marivivens sp. (EM8-6)	EM8-6A	Whelk egg casing (FL)	No	No	
	EM8-6B		No	No	
	EM8-6C		No	No	
	EM8-6D		No	No	
	EM8-6E		No	No	
	EM8-6F		No	No	
	EM8-6G		No	No	
	EM8-6H		No	No	
Sinorhizobium sp. (EM9-1)	EM9-1A	FL Moon Snail	No	No	
	EM9-1B		No	No	
	EM9-1C		No	No	
	EM9-1D		No	No	
	EM9-1E		No	No	
	EM9-1F		No	No	
	EM9-1G		No	No	
	EM9-1H		No	No	
Paracoccus sp. (EM9-2)	EM9-2A	FL Moon Snail	No	No	
	EM9-2B		No	No	
	EM9-2C		No	No	
	EM9-2D		No	No	
	EM9-2E		No	No	
	EM9-2F		No	No	
	EM9-2G		No	No	
	EM9-2H		No	No	
Lysinibacillus sp. (EM9-3)	EM9-3A	Sergeant Major Eggs (New England Aquarium)	No	No	
	EM9-3B		No	No	
	EM9-3C		No	No	
	EM9-3D		No	No	
	EM9-3E		No	No	
	EM9-3F		No	No	
	EM9-3G		No	No	
	EM9-3H		No	No	
Lysinibacillus sp. (EM9-4)	EM9-4A	Crown conch egg	No	No	
	EM9-4B		No	No	
	EM9-4C		No	No	

	EM9-4D	casing (FL)	No	No	
	EM9-4E		No	No	
	EM9-4F		No	No	
	EM9-4G		No	No	
	EM9-4H		No	No	
Euzebyella sp. (EM9-5)	EM9-5A	Whelk egg casing (FL)	No	No	
	EM9-5B		No	No	
	EM9-5C		No	No	
	EM9-5D		No	No	
	EM9-5E		No	No	
	EM9-5F		No	No	
	EM9-5G		No	No	
	EM9-5H		No	No	
Maribacter sp. (EM9-6)	EM9-6A	Banded tulip egg casing (FL)	No	No	
	EM9-6B		Yes (*P. aeruginosa*)	No	
	EM9-6C		No	No	
	EM9-6D		No	No	
	EM9-6E		No	No	
	EM9-6F		No	No	
	EM9-6G		No	No	
	EM9-6H		No	No	
Siansivirga sp. (EM10-1)	EM10-1A	FL Moon Snail	No	No	
	EM10-1B		No	No	
	EM10-1C		No	No	
	EM10-1D		No	No	
	EM10-1E		No	No	
	EM10-1F		No	No	
	EM10-1G		No	No	
	EM10-1H		No	No	
Paenibacillus sp. (EM10-2)	EM10-2A	FL Moon Snail	No	No	
	EM10-2B		No	No	
	EM10-2C		No	No	
	EM10-2D		No	No	
	EM10-2E		No	No	
	EM10-2F		No	No	
	EM10-2G		No	No	
	EM10-2H		No	No	
Paenibacillus sp. (EM10-3)	EM10-3A	Sergeant Major Eggs	No	No	
	EM10-3B		No	No	
	EM10-3C		No	No	

	EM10-3D	(New England Aquarium)	No	No	
	EM10-3E		No	No	
	EM10-3F		No	No	
	EM10-3G		No	No	
	EM10-3H		No	No	
Tenacibaculum sp. (EM10-4)	EM10-4A	Banded tulip egg casing (FL)	No	No	
	EM10-4B		No	No	
	EM10-4C		No	No	
	EM10-4D		No	No	
	EM10-4E		No	No	
	EM10-4F		No	No	
	EM10-4G		No	No	
	EM10-4H		No	No	
Muricauda sp. (EM10-5)	EM10-5A	FL Gastropod Egg Casing	No	No	
	EM10-5B		No	No	
	EM10-5C		No	No	
	EM10-5D		No	No	
	EM10-5E		No	No	
	EM10-5F		No	No	
	EM10-5G		No	No	
	EM10-5H		No	No	
Labrenzia sp. (EM10-6)	EM10-6A	Crown conch egg casing (FL)	No	No	
	EM10-6B		No	No	
	EM10-6C		No	No	
	EM10-6D		No	No	
	EM10-6E		No	No	
	EM10-6F		No	No	
	EM10-6G		No	No	
	EM10-6H		Yes (*S. aureus*)	No	No
Bacillus sp. (EM11-1)	EM11-1A	FL Moon Snail	No	No	
	EM11-1B		No	No	
	EM11-1C		No	No	
	EM11-1D		No	No	
	EM11-1E		No	No	
	EM11-1F		No	No	
	EM11-1G		No	No	
	EM11-1H		No	No	
Bacillus sp. (EM11-2)	EM11-2A	FL Moon Snail	No	No	
	EM11-2B		No	No	
	EM11-2C		No	No	
	EM11-2D		No	No	

	EM11-2E		No	No	
	EM11-2F		No	No	
	EM11-2G		Yes (*S. aureus*)	No	No
	EM11-2H		No	No	
Bacillus sp. (EM11-3)	EM11-3A	FL Moon Snail	No	No	
	EM11-3B		Yes (*P. aeruginosa*)	No	
	EM11-3C		No	No	
	EM11-3D		No	No	
	EM11-3E		No	No	
	EM11-3F		Yes (*S. aureus*)	No	No
	EM11-3G		No	No	
	EM11-3H		No	No	
Pseudoalteromonas piscicida (EM11-4)	EM11-4A	FL Moon Snail	No	No	
	EM11-4B		No	No	
	EM11-4C		No	No	
	EM11-4D		No	No	
	EM11-4E		Yes (*S. aureus*)	No	Yes
	EM11-4F		No	No	
	EM11-4G		No	No	
	EM11-4H		Yes (*S. aureus*)	No	Yes
Paracoccus sp. (EM11-5)	EM11-5A	FL Moon Snail	No	No	
	EM11-5B		No	No	
	EM11-5C		No	No	
	EM11-5D		No	No	
	EM11-5E		No	No	
	EM11-5F		No	No	
	EM11-5G		No	No	
	EM11-5H		No	No	
Bacillus sp. (EM11-6)	EM11-6A	FL Moon Snail	No	No	
	EM11-6B		No	No	
	EM11-6C		No	No	
	EM11-6D		No	No	
	EM11-6E		No	No	
	EM11-6F		No	No	
	EM11-6G		Yes (*S. aureus*)	No	Yes
	EM11-6H		No	No	
Pseudoalteromonas sp. (EM12-1)	EM12-1A	FL Moon Snail	No	No	
	EM12-1B		No	No	
	EM12-1C		No	No	
	EM12-1D		No	No	

	EM12-1E		Yes (*S. aureus*)	No	Yes
	EM12-1F		No	No	
	EM12-1G		No	No	
	EM12-1H		No	No	
Marinomonas sp. (EM12-2)	EM12-2A	FL Moon Snail	No	No	
	EM12-2B		No	No	
	EM12-2C		No	No	
	EM12-2D		No	No	
	EM12-2E		No	No	
	EM12-2F		No	No	
	EM12-2G		No	No	
	EM12-2H		Yes (*S. aureus*)	No	Yes
Ficitbacillus, sp. (EM12-3)	EM12-3A	FL Moon Snail	No	No	
	EM12-3B		No	No	
	EM12-3C		No	No	
	EM12-3D		No	No	
	EM12-3E		Yes (*S. aureus*)	Yes (*S. aureus*)	Yes
	EM12-3F		No	No	
	EM12-3G		No	No	
	EM12-3H		No	No	
Pseudomonas sp. (EM12-4)	EM12-4A	FL Moon Snail	No	No	
	EM12-4B		No	No	
	EM12-4C		No	No	
	EM12-4D		No	No	
	EM12-4E		No	No	
	EM12-4F		No	No	
	EM12-4G		Yes (*S. aureus*)	No	Yes
	EM12-4H		No	No	
Fictibacillus sp. (EM12-5)	EM12-5A	FL Moon Snail	Yes (*S. aureus*)	No	Yes
	EM12-5B		No	No	
	EM12-5C		No	No	
	EM12-5D		Yes (*S. aureus*)	No	Yes
	EM12-5E		Yes (*S. aureus*)	No	Yes
	EM12-5F		No	No	
	EM12-5G		No	No	
	EM12-5H		No	No	
Tropicibacter sp. (EM12-6)	EM12-6A	Whelk egg casing (FL)	No	No	
	EM12-6B		No	No	
	EM12-6C		No	No	
	EM12-6D		No	No	

	EM12-6E		Yes (*S. aureus*)	No	Yes
	EM12-6F		Yes (*S. aureus*)	No	Yes
	EM12-6G		Yes (*S. aureus*)	No	Yes
	EM12-6H		Yes (*P. aeruginosa*)	No	
Bacillus sp. (EM13-1)	EM13-1A	FL Moon Snail	No	No	
	EM13-1B		No	No	
	EM13-1C		No	No	
	EM13-1D		No	No	
	EM13-1E		No	No	
	EM13-1F		No	No	
	EM13-1G		No	No	
	EM13-1H		Yes (*S. aureus*)	No	Yes
Fictibacillus sp. (EM13-2)	EM13-2A	FL Moon Snail	No	No	
	EM13-2B		No	No	
	EM13-2C		No	No	
	EM13-2D		No	No	
	EM13-2E		Yes (*S. aureus*)	No	Yes
	EM13-2F		No	No	
	EM13-2G		No	No	
	EM13-2H		No	No	
Pseudoalteromonas sp. (EM13-3)	EM13-3A	FL Moon Snail	No	No	
	EM13-3B		No	No	
	EM13-3C		No	No	
	EM13-3D		No	No	
	EM13-3E		No	No	
	EM13-3F		No	No	
	EM13-3G		No	No	
	EM13-3H		No	No	
Shima sp. (EM13-4)	EM13-4A	Crown conch egg casing (FL)	No	No	
	EM13-4B		No	No	
	EM13-4C		No	No	
	EM13-4D		No	No	
	EM13-4E		No	No	
	EM13-4F		No	No	
	EM13-4G		No	No	
	EM13-4H		Yes (*P. aeruginosa*)	No	
Aquimarina sp. (EM13-5)	EM13-5A	Banded tulip egg	No	No	
	EM13-5B		No	No	
	EM13-5C		No	No	

	EM13-5D	casing (FL)	No	No	
	EM13-5E		No	No	
	EM13-5F		No	No	
	EM13-5G		Yes (*S. aureus*) and Yes (*P. aeruginosa*)	No	No
	EM13-5H		No	No	
Tenacibaculum sp. (EM13-6)	EM13-6A	FL Gastropod Egg Casing	No	No	
	EM13-6B		No	No	
	EM13-6C		No	No	
	EM13-6D		No	No	
	EM13-6E		No	No	
	EM13-6F		Yes (*S. aureus*)	Yes (*S. aureus*)	Yes
	EM13-6G		No	No	
	EM13-6H		No	No	
Pseudoalteromonas sp. (EM14-1)	EM14-1A	FL Moon Snail	No	No	
	EM14-1B		No	No	
	EM14-1C		No	No	
	EM14-1D		No	No	
	EM14-1E		No	No	
	EM14-1F		Yes (*S. aureus*)	No	Yes
	EM14-1G		Yes (*S. aureus*)	No	Yes
	EM14-1H		No	No	
Pseudoalteromonas sp. (EM14-2)	EM14-2A	FL Moon Snail	No	No	
	EM14-2B		No	No	
	EM14-2C		No	No	
	EM14-2D		No	No	
	EM14-2E		Yes (*S. aureus*)	No	Yes
	EM14-2F		No	No	
	EM14-2G		No	No	
	EM14-2H		No	No	
Pseudoalteromonas sp. (EM14-3)	EM14-3A	FL Moon Snail	No	No	
	EM14-3B		No	No	
	EM14-3C		No	No	
	EM14-3D		No	No	
	EM14-3E		No	No	
	EM14-3F		No	No	
	EM14-3G		No	No	
	EM14-3H		Yes (*S. aureus*)	No	Yes
Pseudomonas sp.	EM14-4A		No	No	

(EM14-4)	EM14-4B	FL Moon Snail	No	No	
	EM14-4C		No	No	
	EM14-4D		No	No	
	EM14-4E		No	No	
	EM14-4F		No	No	
	EM14-4G		No	No	
	EM14-4H		No	No	
Bacillus sp. (EM14-5)	EM14-5A	FL Moon Snail	No	No	
	EM14-5B		No	No	
	EM14-5C		No	No	
	EM14-5D		Yes (*S. aureus*)	No	Yes
	EM14-5E		No	No	
	EM14-5F		Yes (*S. aureus*)	No	Yes
	EM14-5G		No	No	
	EM14-5H		No	No	
Aliiroseovarius sp. (EM14-6)	EM14-6A	Crown conch egg casing (FL)	No	No	
	EM14-6B		No	No	
	EM14-6C		No	No	
	EM14-6D		No	No	
	EM14-6E		No	No	
	EM14-6F		No	No	
	EM14-6G		No	No	
	EM14-6H		No	No	
Bacillus sp. (EM15-1)	EM15-1A	FL Moon Snail	No	No	
	EM15-1B		No	No	
	EM15-1C		No	No	
	EM15-1D		No	No	
	EM15-1E		No	No	
	EM15-1F		No	No	
	EM15-1G		No	No	
	EM15-1H		No	No	
Vibrio sp. (EM15-2)	EM15-2A	FL Moon Snail	No	No	
	EM15-2B		No	No	
	EM15-2C		No	No	
	EM15-2D		No	No	
	EM15-2E		No	No	
	EM15-2F		No	No	
	EM15-2G		No	No	
	EM15-2H		No	No	
Bacillus sp. (EM15-3)	EM15-3A	FL Moon Snail	No	No	
	EM15-3B		No	No	

	EM15-3C		No	No	
	EM15-3D		No	No	
	EM15-3E		No	No	
	EM15-3F		No	No	
	EM15-3G		No	No	
	EM15-3H		No	No	
Paracoccus sp. (EM15-4)	EM15-4A	FL Moon Snail	No	No	
	EM15-4B		No	No	
	EM15-4C		No	No	
	EM15-4D		No	No	
	EM15-4E		No	No	
	EM15-4F		No	No	
	EM15-4G		No	No	
	EM15-4H		No	No	
Paracoccus sp. (EM15-5)	EM15-5A	FL Moon Snail	No	No	
	EM15-5B		No	No	
	EM15-5C		No	No	
	EM15-5D		No	No	
	EM15-5E		No	No	
	EM15-5F		No	No	
	EM15-5G		No	No	
	EM15-5H		No	No	
Isoptericola sp. (EM15-6)	EM15-6A	FL Moon Snail	No	No	
	EM15-6B		No	No	
	EM15-6C		No	No	
	EM15-6D		No	No	
	EM15-6E		No	No	
	EM15-6F		No	No	
	EM15-6G		No	No	
	EM15-6H		No	No	
Bacillus sp. (EM16-1)	EM16-1A	FL Moon Snail	No	No	
	EM16-1B		No	No	
	EM16-1C		No	No	
	EM16-1D		No	No	
	EM16-1E		No	No	
	EM16-1F		No	No	
	EM16-1G		No	No	
	EM16-1H		No	No	
Isoptericola sp. (EM16-2)	EM16-2A	FL Moon Snail	No	No	
	EM16-2B		No	No	
	EM16-2C		No	No	

	EM16-2D		No	No	
	EM16-2E		No	No	
	EM16-2F		No	No	
	EM16-2G		No	No	
	EM16-2H		No	No	
Paenibacillus sp. (EM725)	EM725-A	PR Moon Snail	No	No	
	EM725-B		No	No	
Lelliottia sp. (EM726)	EM726-A	PR Moon Snail	No	No	
	EM726-B		No	No	
Bacillus sp. (EM727)	EM727-A	PR Moon Snail	No	No	
	EM727-B		No	No	
Bacillus sp. (EM728)	EM728-A	PR Moon Snail	No	No	
	EM728-B		No	No	
Exiguobacterium sp. (EM729)	EM729-A	PR Moon Snail	Yes (*S. aureus*)	No	No
	EM729-B			Yes (*S. aureus*)	No
Exiguobacterium sp. (EM730)	EM730-A	PR Moon Snail	No	No	
	EM730-B		No	No	
Kosakonia sp. (EM731)	EM731-A	PR Moon Snail	No	No	
	EM731-B		No	No	
Microbacterium sp. (EM732)	EM732-A	PR Moon Snail	No	No	
	EM732-B		No	No	
Bacillus sp. (EM733)	EM733-A	PR Moon Snail	No	No	
	EM733-B		No	No	
Aurantiacibacter sp. (EM734)	EM734-A	PR Moon Snail	No	No	
	EM734-B		No	No	
Alkalihalobacillus sp. (EM735)	EM735-A	PR Moon Snail	No	No	
	EM735-B		No	No	

Aureimonas sp. (EM736)	EM736-A	PR Moon Snail	No	No	
	EM736-B		No	No	
Staphylococcus sp. (EM737)	EM737-A	PR Moon Snail	Yes (*S. aureus*) and Yes (*P. aeruginosa*)	No	No
	EM737-B			No	No
Microbacterium sp. (EM738)	EM738-A	PR Moon Snail	No	No	
	EM738-B		No	No	
Unknown (EM739)	EM739-A	PR Moon Snail	Yes (*S. aureus*)	No	No
	EM739-B			No	No
Bacillus sp. (EM740)	EM740-A	PR Moon Snail	Yes (*S. aureus*)	No	No
	EM740-B			Yes (*S. aureus*)	No
Unknown (EM741)	EM741-A	PR Moon Snail	No	No	
	EM741-B		No	No	
Unknown (EM742)	EM742-A	PR Moon Snail	No	No	
	EM742-B		No	No	
Rhodobacter sp. (EM743)	EM743-A	PR Moon Snail	No	No	
	EM743-B		No	No	
Curtobacterium sp. (EM744)	EM744-A	PR Moon Snail	No	No	
	EM744-B		No	No	
Microbacterium sp. (EM745)	EM745-A	PR Moon Snail	No	No	
	EM745-B		No	No	
Curtobacterium sp. (EM746)	EM746-A	PR Moon Snail	No	No	
	EM746-B		No	No	
Cytobacillus sp. (EM747)	EM747-A	PR Moon Snail	No	No	
	EM747-B		No	No	

Pseudomonas sp. (EM748)	EM748-A	PR Moon Snail	No	No	
	EM748-B		No	No	
Bacillus sp. (EM749)	EM749-A	PR Moon Snail	No	No	
	EM749-B		No	No	
Unknown (EM750)	EM750-A	PR Moon Snail	No	No	
	EM750-B		No	No	
Paenibacillus sp. (EM751)	EM751-A	PR Moon Snail	No	No	
	EM751-B		No	No	
Bacillus sp. (EM752)	EM752-A	PR Moon Snail	Yes (*P. aeruginosa*)	No	
	EM752-B			No	
Acinetobacter sp. (EM753)	EM753-A	PR Moon Snail	No	No	
	EM753-B		No	No	
Unknown (EM754)	EM754-A	PR Moon Snail	No	No	
	EM754-B		No	No	
Exiguobacterium sp. (EM755)	EM755-A	PR Moon Snail	No	No	
	EM755-B		No	No	
Curtobacterium sp. (EM756)	EM756-A	PR Moon Snail	No	No	
	EM756-B		No	No	
Bacillus sp. (EM757)	EM757-A	PR Moon Snail	No	No	
	EM757-B		No	No	
Unknown (EM758)	EM758-A	PR Moon Snail	Yes (*S. aureus*)	No	No
	EM758-B			No	No
Bacillus sp. (EM759)	EM759-A	PR Moon Snail	No	No	
	EM759-B		No	No	
Curtobacterium sp.	EM760-A		No	No	

(EM760)	EM760-B	PR Moon Snail	No	No	
Staphylococcus sp. (EM761)	EM761-A	PR Moon Snail	No	No	
	EM761-B		No	No	
Staphylococcus sp. (EM762)	EM762-A	PR Moon Snail	Yes (*S. aureus*)	No	No
	EM762-B			No	No
Unknown (EM763)	EM763-A	PR Moon Snail	No	No	
	EM763-B		No	No	
Streptomyces sp. (EM764)	EM764-A	PR Moon Snail	No	No	
	EM764-B		No	No	
Unknown (EM765)	EM765-A	PR Moon Snail	No	No	
	EM765-B		No	No	
Unknown (EM766)	EM766-A	PR Moon Snail	No	No	
	EM766-B		No	No	
Unknown (EM767)	EM767-A	PR Moon Snail	No	No	
	EM767-B		No	No	
Rossellomorea sp. (EM768)	EM768-A	PR Moon Snail	No	No	
	EM768-B		No	No	
Fictibacillus sp. (EM769)	EM769-A	PR Moon Snail	No	No	
	EM769-B		No	No	
Bacillus sp. (EM770)	EM770-A	PR Moon Snail	No	No	
	EM770-B		No	No	
Ruegeria sp. (EM771)	EM771-A	PR Moon Snail	No	No	
	EM771-B		No	No	
Ruegeria sp. (EM772)	EM772-A	PR Moon Snail	No	No	
	EM772-B		No	No	

Ruegeria sp. (EM773)	EM773-A	PR Moon Snail	No	No	
	EM773-B		No	No	
Ruegeria sp. (EM774)	EM774-A	PR Moon Snail	No	No	
	EM774-B		No	No	
Micromonospora sp. (EM775)	EM775-A	PR Moon Snail	No	No	
	EM775-B		No	No	
Oceanobacillus sp. (EM776)	EM776-A	PR Moon Snail	No	No	
	EM776-B		No	No	
Ruegeria sp. (EM777)	EM777-A	PR Moon Snail	No	No	
	EM777-B		No	No	

**Table S2:** Strains and precursor masses of nodes in the GNPS cluster containing 136 Da neutral loss				
	Fraction code	Biofilm (staph)	Antimicrobial (staph)	Precursor Mass
1	EM12-2	yes	no	386.172, 422.133, 427.149, 446.139, 495.224, 509.24
2	EM14-2	yes	no	386.172, 400.211, 446.139, 464.151, 520.174
3	EM11-4	yes	no	386.172, 400.211, 495.224, 509.24
4	EM12-1	yes	no	386.172, 400.211, 495. 224, 509.24
5	EM12-4	yes	no	386.037, 386.172, 400.211, 446.139, 464.151, 520.174
6	EM11-6	yes	no	386.037, 386.172, 395.159, 427.149
7	EM11-5	no	yes	386.037, 386.172, 400.211, 427.149, 495.224, 509.24
8	EM13-3	no	yes	386.037, 386.172, 400.211, 422.133, 446.139, 464.151, 495.224, 509.24, 520.174
9	EM15-2	no	yes	386.172
10	EM13-4	no	yes	386.172
11	EM5-4	no	no	386.172, 446.139, 464.151
12	EM12-3	yes	no	386.172, 400.211, 464.151, 509.24
13	EM12-6	yes	yes	386.172
14	EM14-1	yes	no	382.172
15	EM15-1	no	yes	395.159, 427.149
16	EM12-5	yes	yes	395.159

Table 3: Media Recipes

YEME	R2A	A-media	RAM	chitin	Marine agar	M63	Artificial Seawater
4 g yeast extract	0.5 g acicase	20 g starch	4 g cornmeal	3 g chitin	5 g peptone	2 g $(NH_4)SO_4$	1:1 mixture of solution A and B
10 g Malt extract	0.5 g yeast extract	5 g peptone	15 g maltose	0.575 g K_2HPO_4	1 g yeast extract	13.6 g KH_2PO_4	
4 g dextrose	0.5 g proteose peptone	10 g dextrose	10 g dextrose	0.375 g $MgSO_4.7H_2O$	0.1 g Ferric citrate	0.5 mg $FeSO_4$ $7H_2O$	**Solution A**
20 g Agar	0.5 g dextrose	5 g yeast	7.5 g pharmamedia	0.275 g KH_2PO_4	19.45 g NaCl	1 mL of 1 M $MgSO_4.7H_2O$	415.2 g NaCl
1 L seawater	0.5 g starch, soluble	5 g $CaCO_3$	5 g primary yeast	7.5 mg $FeSO_4.7H_2O$	8.8 g $MgCl_2$	10 mL of 20% glucose (water)	69.5 g Na_2SO_4
	0.3 g dipotassium hydrogen phosphate	1 L seawater	1 L seawater	0.75 mg $MnCl_2.4H_2O$	3.24 g $NaSO_4$	0.1 mL of 0.5% thiamine	11.74 g KCl
	0.024 g magnesium sulphate			0.75 mg $ZnSO_4.7H_2O$	1.8 g $CaCl_2$	5 mL of 20% casamino acids	3.4 g $NaHCO_3$
	0.3 g sodium pyruvate			1 L seawater	0.55 g KCl_2	1 L MilliQ water	1.7 g KBr
	1 L seawater				0.16 g $NaHCO_3$		0.45 g H_3BO_3
					0.08 g KBr_2		0.054 g NaF
					34 mg $SrCl_2$		10 L DI water
					22.0 mg H_3BO_3		
					4.0 mg Na_2SiO_3		**Solution B**
					2.4 mg NaF		187.9 g $MgCl_3$.6H2O
					1.6 mg NH_4NO_3		22.7 g $CaCl_2.2H_2O$
					8.0 mg Na_2HPO_4		0.428 g $SrCl_2.6H_2O$
					15 g agar		10 L DI water
					1 L DI water		

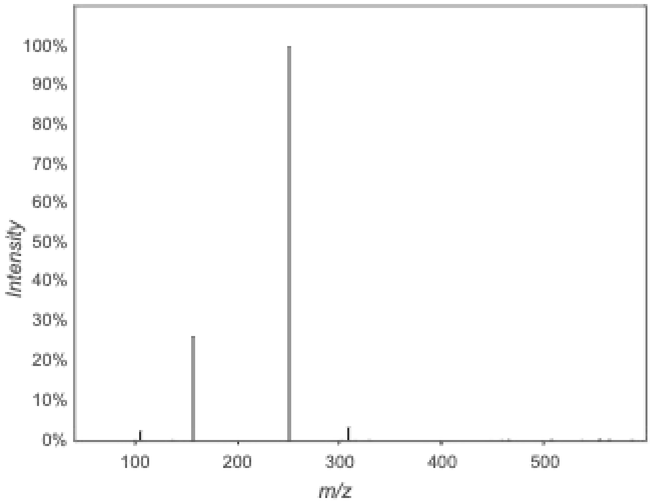

Figure S12: Fragmentation of precursor mass 386.037 m/z

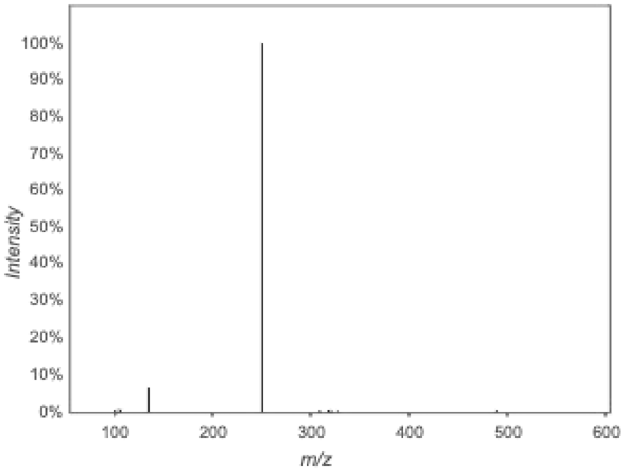

Figure S13: Fragmentation of precursor mass 386.172 m/z

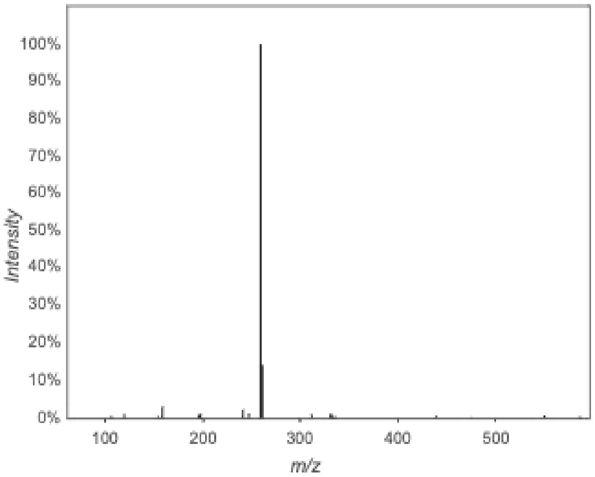

Figure S14: Fragmentation of precursor mass 395.159 m/z

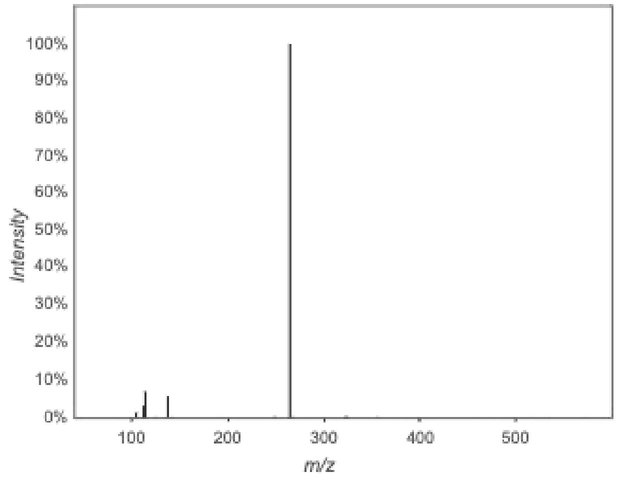

Figure S15: Fragmentation of precursor mass 400.211 m/z

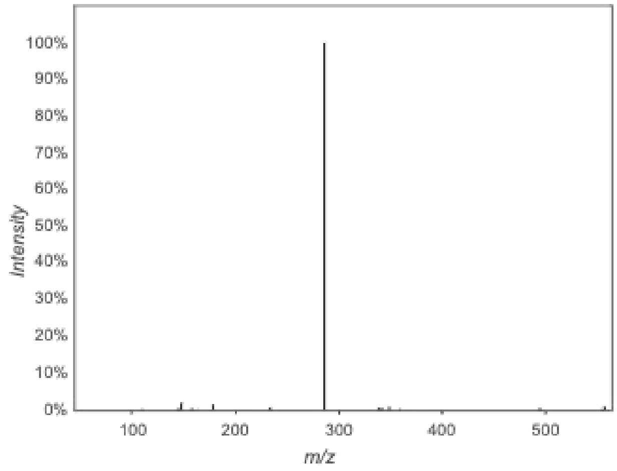

Figure S16: Fragmentation of precursor mass 422.133 m/z

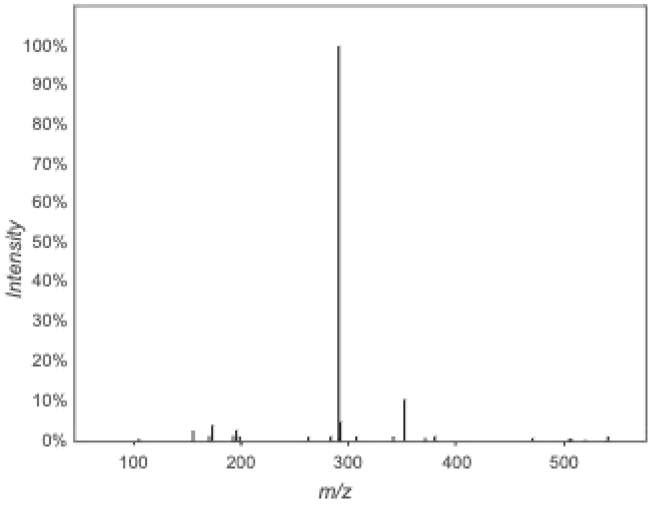

Figure S17: Fragmentation of precursor mass 427.149 m/z

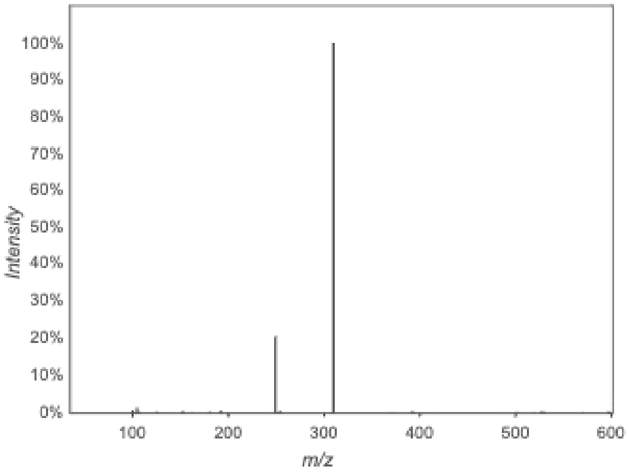

Figure S18: Fragmentation of precursor mass 446.139 m/z

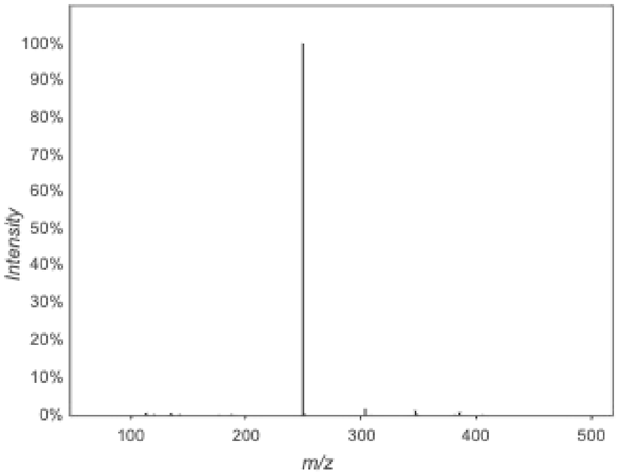

Figure S19: Fragmentation of precursor mass 464.151 m/z

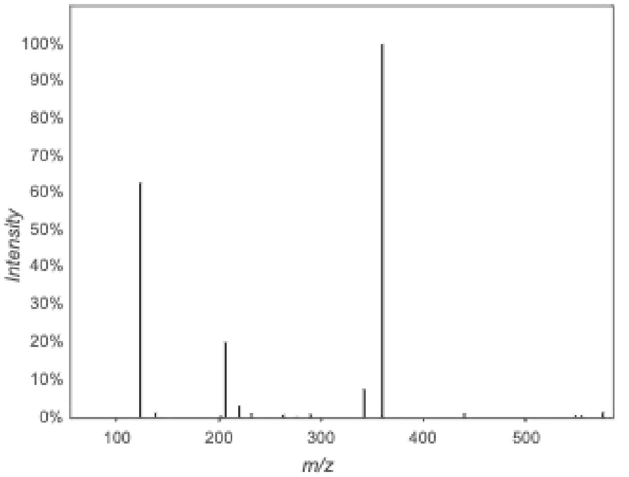

Figure S20: Fragmentation of precursor mass 495.224 m/z

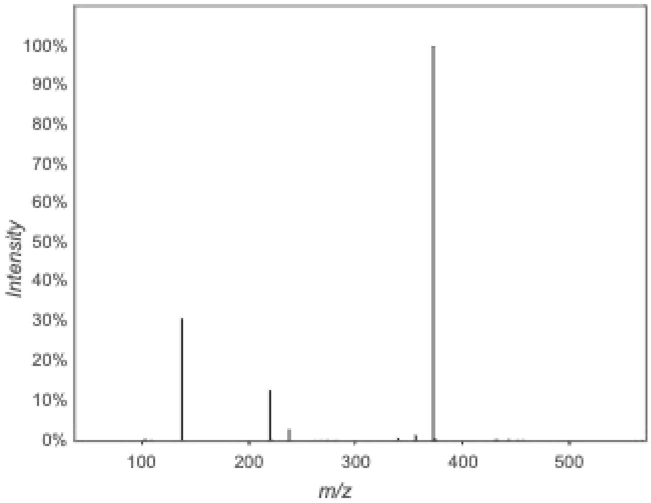

Figure S21: Fragmentation of precursor mass 509.240 m/z

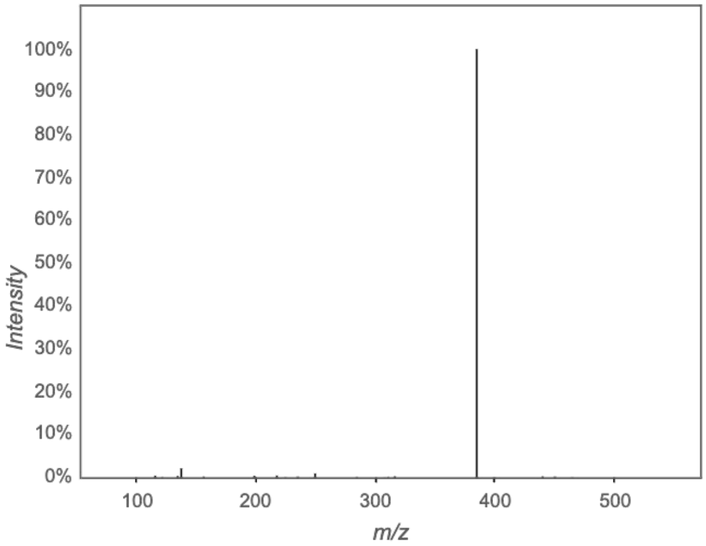

Figure S22: Fragmentation of precursor mass 520.174 m/z

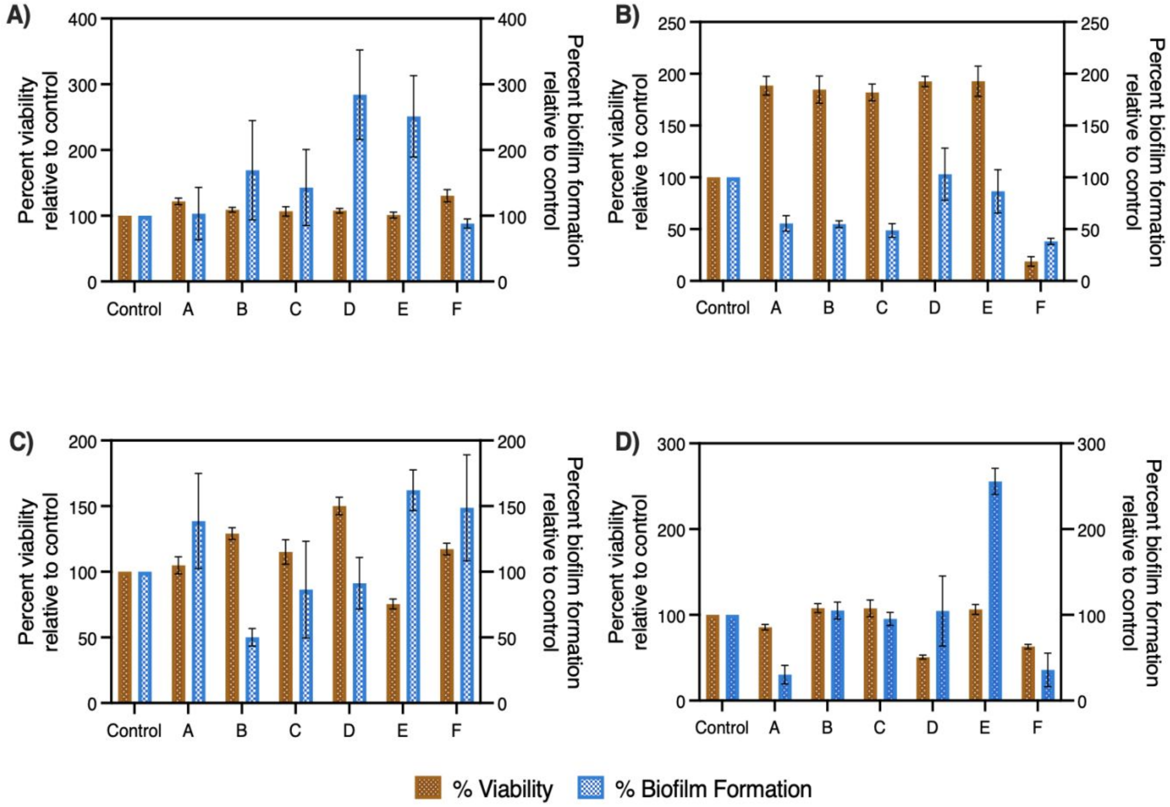

Figure S23: Biofilm inhibition by moon snail egg mass collars chemical fractions collected from Florida and Puerto Rico. **A)** None of the fractions from Florida inhibited *P. aeruginosa* biofilms, **B)** three fractions from Florida inhibited *S. aureus* biofilms without impacting growth viability, **C)** one fraction from Puerto Rico inhibited *P. aeruginosa* biofilms, and **D)** one fraction from Puerto Rico inhibited *S. aureus* biofilms.

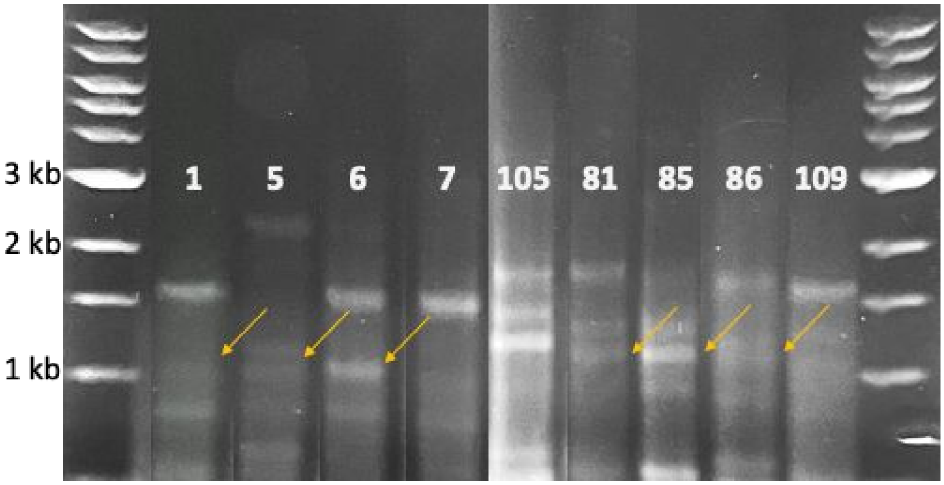

Figure S24: PCR amplification of NRPS adenylation domain from egg collar DNA extracts. Orange arrows indicate adenylation domain band.

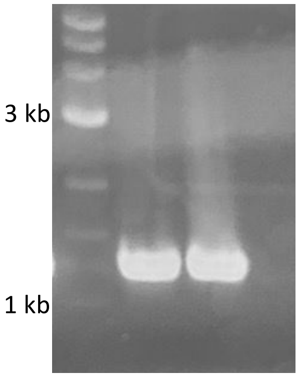

Figure S25: PCR amplification of NRPS-A domain for sequencing. The product of lane 6 in **Figure S24** was gel purified and reamplified using the same PCR amplification. This product was sequenced using Illumina sequencing.

Table S4: Protein matches and e-values for sequences obtained from Illumina sequencing.

**Sequence A**			
**Source organism**	**E-value (blastn)**	**Protein Match**	**E-value (blastx)**
Pseudomonas piscicida	$1e^{-153}$	Non-Ribosomal Peptide Synthetase	$2e^{-58}$
**Sequence**	ATGAGCGGTTAGTACAGCAAGTGCTATGGCTAGCAAAACGCCTTA<br>GAGTCAAAACTTCCGTCGATGATGGTACCGTATTGCTAGCACTGCC<br>TCGTAGCCAATATACAGTGATTGCAATGTGGGCCGTATTTGCTGCG<br>CAGCACCGCTTCGTTTTTATTGATAGCGATGCTCCTTCTGCGCGTA<br>ATCGTAGTATCATCGCTGATGCTAAGCCATGCCTAGCATTGGTGGA<br>TGATAGTAGTGAAGGGCTAGCTGAGATCCGCGACGCTGGCGTTAC<br>ACAGCTTAATATTGCAAGCGAACTTGCAAATGCCCATTCTTGAAGC<br>CGCGGTG		
**Sequence B**			
**Source organism**	**E-value (blastn)**	**Protein Match**	**E-value (blastx)**
Pseudomonas piscicida	$1e^{-139}$	EAL Domain Containing Protein	$4e^{-56}$
**Sequence**	ATGAGCGGTTAGTACAGCAACTGTTTGATGCCGAACGCAACGATG<br>AGCTTGCGGTAGTGTTTTTTATTGATTTAGATGATTTTAAAAGGATC<br>AATGATTCACTCGGACACGAAACTGGGGATCAAATTCTCATCGCTT<br>CAGGAGAAAGGCTCAAGCAATCGCTACGTGAGCAAGATACCGTGT<br>CACGTCTTAGTGGTGATGAGTTTATTGTGTTGATGGGAGGGTTTAA<br>GCACACTGCAGACATTACCTCTGTTGCCGATATGCTCATCAAGCTT<br>TTTCACCATCCCATTCTTGAAGCCGCGGTG		
**Sequence C**			
**Source organism**	**E-value (blastn)**	**Protein Match**	**E-value (blastx)**
Pseudomonas piscicida	$1e^{-43}$	No Significant Matches	------
**Sequence**	ATGAGCGGTTAGTACAGCAGAGATCCAGTTCTGTTTCCTGGCTGTT<br>ATGTGTTTCAGACATCTTATTCTGTTTCCTGGCAGTATGTGTGTGTG<br>GGATATCTCATTCTGTTTCCTGGCAATGTCACAGAAATCTCATTCTT<br>GAAGCCGCGGTGATACACTGTGTTACATTCTTGAAGCCGCGGTGA<br>TACACTGTGTTACATGACAGCTATTAGCTATGAGCGGTTAGTACAG<br>CAAATACAAACTAACCTAGATTAGACGCTGGTAAATTTAGCAACCG<br>TTTTATGGTCTGCTTTTTAGACGCACTTTACTTGCTACTGGCCTTTA<br>TTGATATGCTTATTTATCCATTCTTGAAGCCGCGGTG		

**Table S5**: Genome assembly and annotation parameters			
**Tool**	**Version**	**Parameters**	**Application**
Porechop	0.2.4	default	Remove residual adaptor sequences
Flye	2.9.2	--asm-coverage 50<br>--genome-size 6000000<br>--nano-hq	*De novo* genome assembly
Circulator	1.5.5	All; 6 hr timeout	Evaluate assembled contigs for circularization.
Bakta	1.8.1	Default	Genome annotation with Bakta database (v. 5.0)
Quast	5.2.0	Default	Record assembly statistics
